# Potential of *Serratia* sp. KF23 in stimulating soybean growth and alleviating the effects of salinity stress in a three-year pot experiment

**DOI:** 10.1007/s11274-026-05055-0

**Published:** 2026-05-30

**Authors:** Iryna Kulkova, Jakub Dobrzyński, Barbara Wróbel

**Affiliations:** Institute of Technology and Life Sciences – National Research Institute, Al. Hrabska 3, Raszyn, 05-090 Poland

**Keywords:** Salinity stress, Halotolerant bacteria, Plant growth-promoting bacteria (PGPB), *Serratia*, Soybean

## Abstract

Soil salinity may constitute a serious limitation to soybean (*Glycine max* L. Merr.) production. In the present study, a halotolerant bacterial strain KF23 was isolated and characterized, and its ability to support soybean growth under salinity stress conditions was evaluated. KF23 exhibited moderate salt tolerance (6% NaCl) and tolerated a wide range of pH (6–10) and temperature (6–42 °C). Subsequently, 16 S rRNA analysis showed that strain KF23 belongs to the genus *Serratia* and is closely related to *Serratia plymuthica*. *Serratia* sp. KF23 produced indole-3-acetic acid, siderophores and NH_3_, exhibited nitrogen fixation and ZnSO_4_ solubilization, as well as proteolytic and β-1,3-glucanase activity. KF23 formed biofilm and exopolysaccharides at 0–6% NaCl. In a three-year pot experiment, soybean plants were grown under 0, 150, and 300 mM NaCl with or without KF23 inoculation. KF23 inoculation improved soybean growth at 150 and 300 mM NaCl, increasing root length (+ 32–39.6%), shoot fresh weight (+ 9.8–45.1%), shoot dry weight (+ 19.1–23.3%), and fresh pod weight (+ 23.3–36.9%) relative to the control. Chlorophyll a and b content was higher in inoculated plants, whereas the levels of malondialdehyde, hydrogen peroxide, and proline were lower, indicating a reduction of osmotic and oxidative stress. Catalase activity increased significantly (+ 157.6% at 0 mM and + 20% at 150 mM NaCl). In the soil, a higher total nitrogen concentration was recorded at 300 mM NaCl (+ 11.4%), and dehydrogenase activity remained stable. KF23 also increased protein content and digestibility of soybean above-ground biomass under salinity conditions. The results indicate that *Serratia* sp. KF23 is a promising plant growth-promoting bacterium improving plant performance and soil quality under salt stress conditions.

**Supplementary Information:**

The online version contains supplementary material available at 10.1007/s11274-026-05055-0.

## Introduction

Salinity is one of the major abiotic stresses that has a serious impact on crop cultivation, limiting plant growth and productivity (Zhao et al. 2021). According to the latest information presented by FAO (2024), salinity has affected nearly 1.4 billion hectares of land, which corresponds to 10.7% of the global land surface. It is predicted that 50% of all arable land will be affected by salinity by 2050 (Wang et al. 2003; Butcher et al. 2016). Approximately 31 million hectares of land throughout Europe are affected by salinity, and this problem occurs mainly in Cyprus, Hungary, Romania, Portugal, Greece, Italy, and in the regions of the Iberian Peninsula (Matuszak-Slamani et al. 2017; Rossini et al. 2024). The intensification of salinity results from two main groups of factors. First, these are anthropogenic factors, including poor cultivation practices such as improper fertilizer use, irrigation with low-quality water, improper drainage or irrigation methods, deforestation, and the removal of deep-rooted vegetation; second, these are climate changes (natural salinity), such as increasing drought and freshwater shortage, and increasing salinity of groundwater and surface waters (Daliakopoulos et al. 2016; Cuevas et al. 2019).

Salinity negatively affects the morphological, biochemical, molecular, and physiological functioning of most crop plants by inducing osmotic stress, ionic toxicity (mainly excess Na⁺ and Cl⁻ and disturbance of K⁺/Na⁺homeostasis), and oxidative stress resulting from the overproduction of reactive oxygen species (ROS) (Kulkova et al. 2023). Under conditions of intensified oxidative stress, increases in the concentrations of hydrogen peroxide (H_2_O_2_), proline, and malondialdehyde (MDA), as well as changes in the activity of antioxidant enzymes such as superoxide dismutase (SOD), catalase (CAT), ascorbate peroxidase (APX), and peroxidase (POD), are observed, reflecting the ability of plants to maintain redox balance (Wang et al. 2016; Singh and Tiwari 2021; Mousavi et al. 2022).

Soybean (*Glycine max* L. Merr.) is one of the most important crop plants in the world due to the high nutritional value of its seeds, its wide use in the food industry, biofuel production, and as a primary source of protein in livestock feed (Sobko et al. 2020; Santos et al. 2019; Pope et al. 2023). Soybeans account for 69% of global protein consumption and 29% of vegetable oil consumption (Wang et al. 2024). Moreover, soybean, as a legume plant, participates in biological nitrogen fixation, which increases its importance in sustainable agriculture systems and reduces dependence on mineral fertilization (Hungria and Mendes 2015). Soybean is moderately tolerant to salt stress; however, high salinity can cause yield losses of up to 40% (Luo et al. 2005; Guan et al. 2014). Ionic imbalance resulting from salinity disrupts normal metabolic processes in soybean, reduces nitrogen metabolism efficiency, impairs photosynthesis, and ultimately leads to oxidative stress through excessive production of reactive oxygen species (ROS) (Hasanuzzaman et al. 2022). Therefore, as a crop of high economic value and significant agricultural importance, soybean represents an important subject of research in the context of mitigating the effects of salinity stress. Consequently, there is a growing need to develop effective and sustainable strategies to enhance soybean tolerance to salinity.

In the last decade, interest has increased in the use of halotolerant and halophilic plant growth-promoting bacteria (PGPB) as a biological tool for alleviating the effects of salinity stress (Yasmin et al. 2020; Orhan and Demirci 2020; Patel et al. 2023). Numerous studies indicate that PGPB can alleviate the negative effects of salinity stress through direct mechanisms: (i) increasing the availability of nutrients (including phosphate, zinc, and potassium solubilization), (ii) biological nitrogen fixation (N_2_) and ammonia (NH_3_) production, (iii) siderophore production, (iv) synthesis of phytohormones and growth regulators such as auxins (e.g., indole-3-acetic acid, IAA), cytokinins, abscisic acid (ABA), gibberellins (GA) and 1-aminocyclopropane-1-carboxylic acid (ACC) deaminase activity, (v) biofilm production and exopolysaccharide (EPS) secretion (Bhat et al. 2020; Kasim et al. 2016). The effect is often better root system development, more efficient uptake of water and nutrients, greater tolerance to osmotic and ionic disturbances, and improvement of the physicochemical and biological properties of soil under salinity stress conditions (Shilev 2020; Tang et al. 2023).

Moreover, PGPB also act through an indirect mechanism consisting in limiting pathogen pressure and strengthening plant resistance. These mechanisms include, among others, the production of antibiotics and siderophores, the synthesis of extracellular enzymes hydrolyzing pathogen structures, competition within the rhizosphere for resources, and the induction of systemic resistance (ISR). In the context of salinity, these actions may be additionally important because abiotic stress often increases plant susceptibility to infections and intensifies yield losses through a cumulative effect (Gupta et al. 2022; Wang et al. 2018; Qin et al. 2016; Choudhary et al. 2016). Despite numerous studies on the use of PGPB under salinity stress conditions, there remains a need to identify new strains with high salinity tolerance, particularly those isolated from agricultural soils not previously exposed to this stress, as they may exhibit significant adaptive potential.

In view of the above, the aim of the present study was (i) to select halotolerant bacteria with plant growth-promoting (PGP) traits, including, among others, biofilm and EPS production, P and Zn solubilization, biological N_2_ fixation, NH_3_ production, IAA synthesis, siderophore production, and hydrolytic enzyme production, and (ii) to assess the effect of the interaction of the selected PGPB strain with soybean (*Glycine max* L. Merr.) on the alleviation of salinity stress in a three-year pot experiment. To the best of our knowledge, this is the first study to present a three-year evaluation of key plant physiological traits and soil properties following the application of bacteria from the genus *Serratia*.

## Materials and methods

### Soil sampling and isolation of PGPB

Bulk soil samples were collected at a depth of 10–20 cm from non-saline arable land located at the experimental station of the Institute of Technology and Life Sciences – National Research Institute (52°08’03.6"N 20°55’10.2"E) in Falenty, Poland. Soil samples were placed in sterile plastic containers and stored at 4 °C for further analyses. The PGPB isolation process was carried out according to standard microbiological procedures using the serial dilution method. Plates were poured with nutrient agar and incubated at 28 ± 2 °C for 24–48 h. Subsequently, single colonies were isolated and transferred onto nutrient agar plates to obtain pure bacterial cultures. Morphological characteristics of distinct colonies of each isolate were observed with respect to different shapes, sizes, margins, surfaces, opacity, and elevation.

### Investigation of tolerance to salt stress, pH, and temperature

To assess the NaCl tolerance potential, all isolated bacterial isolates were cultured at 28 ± 2 °C for 24 h in lysogeny broth (LB). Then, 20 µl of culture was introduced into LB medium supplemented with 0–8% NaCl (Singh et al. 2015). Cultures were grown at 28 ± 2 °C for 24, 48, and 72 h, and culture growth was determined spectrophotometrically at 600 nm (BioTek Epoch 2 Reader, USA). The isolate selected for further characterization was named KF23. The halotolerant isolate was stored at -80 °C until use. The tolerance of strain KF23 to different pH values was evaluated by culturing the strain in LB medium containing different pH values (4–11) (Reang et al. 2022). Incubation was conducted at 28 ± 2 °C for 24, 48, and 72 h. Microbial growth was assessed spectrophotometrically at 600 nm. Temperature tolerance was tested by incubating KF23 at 5–45 °C for 24–72 h.

### Amplification of the bacterial 16 S rRNA gene, sequence analysis, and phylogenetic tree construction

Isolate KF23 was identified based on 16 S rRNA gene sequence analysis. Genomic DNA was isolated using the Genomic Mini Kit (A&A Biotechnology, Poland). For amplification of the 16 S rRNA gene, polymerase chain reaction (PCR; Bio-Rad) was performed using standard primers: 27 F (5′-AGAGTTTGATCCTGGCTCAG-3′) and 1492R (5′-AAGGAGGTGATCCAGCCGCA-3′), under previously described standard conditions (Edwards et al. 1989). Sanger sequencing of the purified PCR product was performed by NEXBIO (Lublin, Poland). The obtained nucleotide sequences were compared with the GenBank database using the NCBI BLAST algorithm, and the results were deposited in the NCBI database. The phylogenetic tree for 16 S rRNA sequence analysis was generated using the neighbor-joining algorithm (Saitou and Nei 1987). Bootstrap analysis of the phylogenetic tree was performed with 1000 replications in Mega 7 software (Felsenstein 1985; Kumar et al. 2016).

### Biofilm and EPS production

The biofilm-forming ability and the dynamics of biofilm formation by isolate KF23 under salinity stress conditions were assessed according to the method of O’Toole and Kolter (1998). Into the wells of a 96-well plate, 10 µL of overnight bacterial culture (OD_600_ = 1) and 190 µL of LB medium with NaCl (0–8%) were introduced. Plates were incubated at 28 ± 2 °C for 24, 48, and 72 h. Control wells contained LB and LB with NaCl, without inoculum. After incubation, the contents of the wells were removed, and to eliminate planktonic cells, wells were washed three times with phosphate-buffered saline (PBS, pH 7.2). Then, cells adhering to the wells were stained with 0.1% crystal violet for 30 min. Excess dye was removed by washing three times with PBS, and the plate was dried at room temperature. The bound dye was dissolved in 99.9% ethanol, and staining intensity was measured spectrophotometrically at 570 nm. The test was performed three times, and data are presented as mean ± standard error from three independent measurements.

Isolate KF23 was inoculated onto EPS medium (g L⁻¹: yeast extract 20 g, K_2_HPO_4_ 15 g, MgSO_4_ 0.2 g, MnSO_4_ 0.015 g, FeSO_4_ 0.0015 g, CaCl_2_ 0.03 g, NaCl 0.015 g, 1.5% agar, sucrose 100 g, agar 18 g, pH 7.5) (Paulo et al. 2012). Plates were incubated for 48 h at 28 ± 2 °C. The presence of a mucoid layer on the medium surface was considered an indicator of EPS production. For quantitative analysis, KF23 was grown in LB with 4% sucrose and NaCl (0–8%) in 100 mL Erlenmeyer flasks at 28 ± 2 °C, 130 rpm for 72 h. EPS was precipitated from the supernatant using pre-chilled ethanol (3:1, v/v) and incubated at 4 °C for 12 h. The EPS precipitate was centrifuged for 20 min at 15,000 × g, dried at 60 °C for 24 h, and the dry mass was determined (Azeredo and Oliveira 1996).

### Phosphate and zinc solubilization

The ability of KF23 to solubilize phosphates was tested qualitatively using the National Botanical Research Institute’s phosphate medium (NBRIP) containing inorganic tricalcium phosphate (Nautiyal 1999). After incubation at 28 ± 2 °C for 7 days, plates were observed for halo zones.

The ability of the studied bacterial isolate to solubilize Zn was evaluated by the point inoculation method on Tris-minimal medium (g L⁻¹: dextrose 10 g, (NH_4_)_2_SO_4_ 1.0 g, MgSO_4_ 0.2 g, KCl 0.2 g, K_2_HPO_4_ 0.1 g), containing insoluble and soluble zinc compounds (ZnO or ZnSO_4_) at a concentration of 0.1% (w/v) and 1.5% agar (Rajawat et al. 2016). Plates were incubated at 28 ± 2 °C for 5 days. The presence of clear/white zones around the colonies, indicating the ability of KF23 to solubilize zinc, was considered a positive result.

### Indole-3-acetic acid (IAA) production

To quantify IAA production, the isolate was grown in LB supplemented with tryptophan (1.0 g L⁻¹) at 28 ± 2 °C for 72 h with shaking at 160 rpm (Bric et al. 1991). The culture was centrifuged at 12,000 × g for 10 min, and 500 µL of supernatant was mixed with 1000 µL of Salkowski reagent and incubated in the dark for 60 min. Absorbance was measured at 530 nm, and IAA concentration was determined using a standard curve (10–100 µg/mL).

### Determination of ammonia (NH_**3**_) production, nitrogen fixation, and siderophore production

The bacterial isolate was inoculated into 10 mL of peptone water and incubated for 72 h at 28 ± 2 °C. After incubation, 0.5 mL of Nessler’s reagent was added (Cappuccino and Sherman 1992). NH_3_ production was detected based on the appearance of a yellow-brown or orange color. In addition, a quantitative assessment of the amount of NH_3_ produced by KF23 was carried out. For this purpose, absorbance was measured at a wavelength of 450 nm using a spectrophotometer and calculated on the basis of an ammonium sulfate standard curve (1–10 µmole mL^− 1^) (Bhattacharyya et al. 2020). The nitrogen-fixing ability of KF23 was examined on nitrogen-free Burk agar medium (Chakraborty and Tribedi 2019). Plates were incubated at 28 ± 2 °C for 5–7 days. Positive activity was confirmed by the formation of bacterial colonies on agar plates. Qualitative evaluation of siderophore production was carried out according to the method of Hu and Xu (2011). Strain KF23 was point-inoculated onto a CAS agar plate. After inoculation, the plate was incubated at 28 ± 2 °C for 5–7 days, and the formation of a yellow or orange zone around the bacterial colony was observed (Arora and Verma 2017).

### Catalase production and hydrolytic enzymes

The catalase test was performed by placing 20 µL of an overnight culture of KF23 on a microscope slide and adding 20 µL of 3% H_2_O_2_; the formation of bubbles within 1 min indicated a positive result (Reiner 2010). The ability of KF23 to produce β-1,3-glucanase was assessed using the medium described by Zhou et al. (2021). After 4 days of incubation at 28 ± 2 °C, the presence of a halo zone around the colony indicated a positive result. Proteolytic activity was assessed on LB agar with 10% skimmed milk. KF23 was point-inoculated on plates and incubated for 2–3 days at 28 ± 2 °C. A clear zone indicated positive protease production (Morris et al. 2012). Cellulolytic activity (CMCase) was assessed by point inoculation of KF23 on minimal salt medium (MSM) supplemented with 1% carboxymethylcellulose (CMC) (Teather and Wood 1982). Plates were incubated for 3–5 days at 28 ± 2 °C, then flooded with 0.02% Congo red solution for 15 min. Clear zones indicated cellulolytic activity.

### Three-year pot experiment

#### Application of the halotolerant isolate KF23 on soybean plants

The research was carried out in Falenty, Poland (52°08’03.6"N 20°55’10.2"E) from the first half of May to the end of July in the years 2023–2025. Pot experiments were conducted in a polytunnel.

**Fig. 1:**
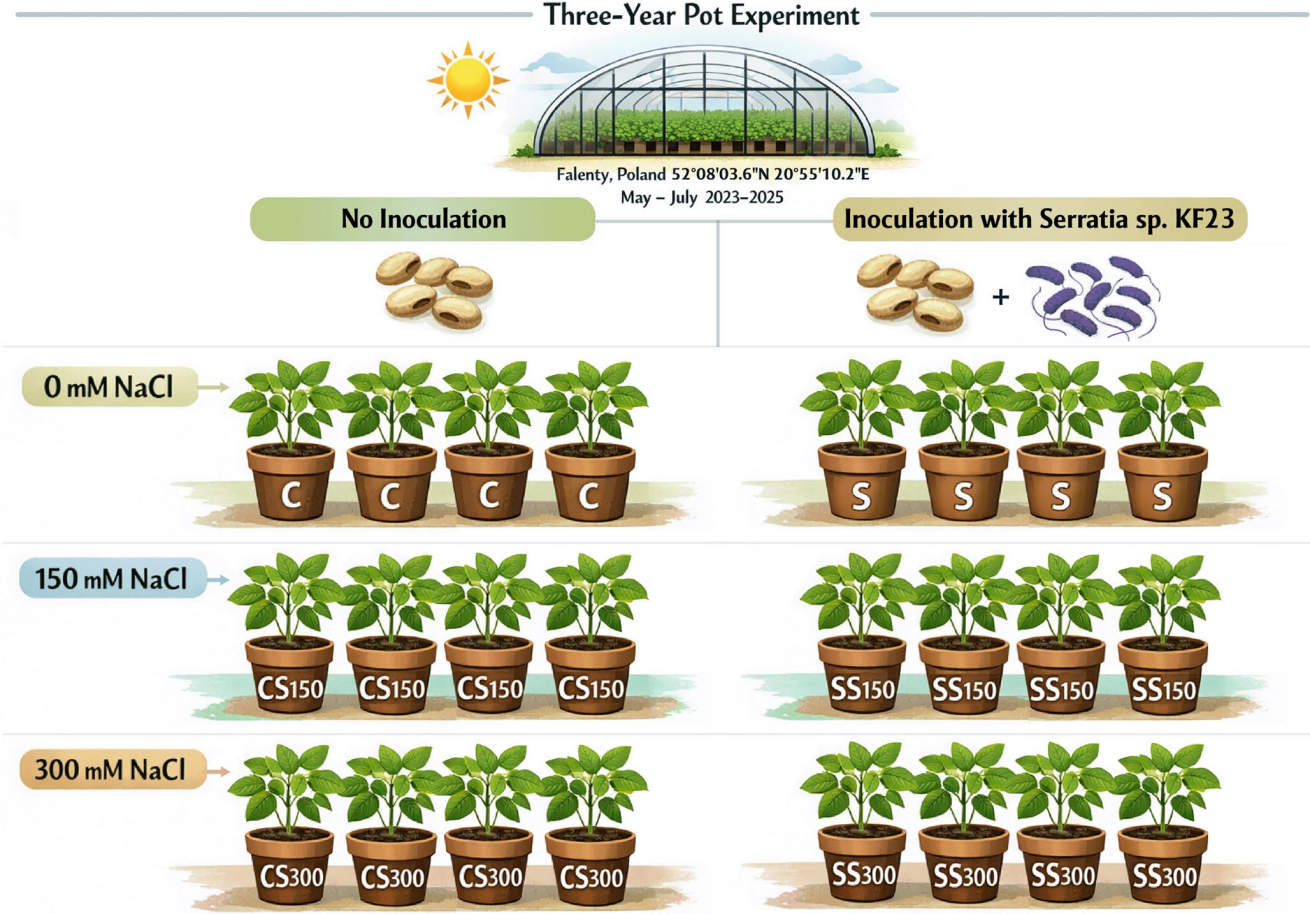
Experimental design of the three-year (2023–2025) pot experiment

The study was carried out in a factorial design including two levels of inoculation with the isolate KF23 (no inoculation and inoculation) and three salinity levels (0, 150, and 300 mM NaCl) applied in the form of a solution for watering plants. Six experimental treatments were established (Fig. 1):

1. C - non-inoculated seeds,
2. S - seeds inoculated with *Serratia* sp. KF23,
3. CS150 - non-inoculated seeds; plants watered with 150 mM NaCl solution,
4. SS150 - seeds inoculated with *Serratia* sp. KF23; plants watered with 150 mM NaCl solution,
5. CS300 - non-inoculated seeds; plants watered with 300 mM NaCl solution,
6. SS300 - seeds inoculated with *Serratia* sp. KF23; plants watered with 300 mM NaCl solution.

The experiment was conducted in a completely randomized factorial design with four replicates for each treatment in each year (a total of 24 pots per year). Each year was treated as an independent experimental cycle, conducted under identical environmental conditions using fresh soil and the same experimental design.

The results of mean temperatures in the polytunnel during soybean growth (2023–2025) are presented in Supplementary Table 1.

### Seed inoculation and treatment application

Soybean seeds (*Glycine max* (L.) Merr., cultivar Albelina) were surface-sterilized in 5% NaOCl solution for 2 min and then in 70% ethanol for another 2 min. After sterilization, the seeds were rinsed six times with autoclaved distilled water and air-dried (Zubair et al. 2019). The bacterial inoculum was prepared by transferring a single colony of isolate KF23 into 5 mL of LB broth and incubating it with shaking at 160 rpm for 24 h at 28 ± 2 °C. Then, the suspension was centrifuged at 12,000 × g for 10 min to obtain a bacterial pellet. The pellets were suspended in sterile 1% carboxymethylcellulose (CMC) solution, serving as a binder. The optical density of the suspension was determined using a densitometer (Biosan Den-1), adjusting it to 0.5 McFarland, which approximately corresponds to a concentration of 1.5 × 10⁸ CFU mL^-1^. Then, under aseptic conditions, surface-sterilized seeds were soaked in the prepared bacterial inoculum for 3 h. In the case of the control, sterile seeds were soaked in sterile 1% CMC (Cherif-Silini et al. 2019). Seeds were sown in bottomless pots, each containing 12 kg of soil. Sandy soil belonging to the Gleysols class was used in the pot study (WRB 2014). Physicochemical properties of the soil prior to the pot experiment (mean for 2023–2025): pH 7.13 ± 0.16, available Ca 2783.45 ± 201.01 mg kg⁻¹ DW, available K 504.76 ± 153.23 mg kg⁻¹ DW, available P 241.60 ± 19.88 mg kg⁻¹ DW, organic carbon (OC) 2.38 ± 0.30% (*n* = 3). Sowing was performed at a rate of 5 seeds per pot, at a depth of 3–4 cm. At the two-leaf development stage, thinning was carried out, leaving 2 plants per pot. From the moment of sowing, all studied plants were watered with tap water (pH 7) in the amount of 400–450 mL every 2–3 days. After 30 days, three salinity levels (0, 150, and 300 mM NaCl) were introduced by watering the plants every 6–7 days with NaCl solution at 400–450 mL per application. To prevent osmotic shock, the NaCl solution was introduced gradually, starting from 50 mM NaCl. No mineral fertilization was applied throughout the experiment.

Each year, the effect of *Serratia* sp. KF23 inoculation under salinity conditions on biometric parameters, photosynthetic pigment content, plant biochemical parameters, and soil physicochemical and enzymatic traits was assessed. Data were recorded 82–83 days after sowing, at the seed maturity stage.

### Biometric parameters

After 82–83 days of plant growth, the following biometric parameters were calculated: fresh and dry weight of roots, shoots, and pods, as well as root length and shoot height of plants. After harvest, soybean roots were washed with distilled water. Root and shoot lengths were measured manually using a ruler. Fresh weight of roots, shoots, and pods was weighed using an analytical balance. Dry weight of roots, shoots, and pods was weighed after drying in an oven at 105 °C to constant weight (Tirry et al. 2021).

### Photosynthetic pigments

The content of chlorophyll *a* (chl *a*), chlorophyll *b* (chl *b*), total chlorophyll (chl tot), and carotenoids was determined quantitatively in fresh samples of upper plant leaves according to the method of Arnon (1949). For this purpose, 0.5 g of plant material was homogenized in 10 mL of chilled 80% acetone. Homogenates were stored for 24 h and then centrifuged at 12,000 × g for 10 min at 4° C. The absorbance of the obtained supernatant was measured spectrophotometrically at wavelengths of 663 nm, 645 nm, and 480 nm. The content of photosynthetic pigments was calculated according to the formulas:

Chl *a* (mg/g FW) = [(12.7 × OD 663) − (2.69 × OD 645)] × V / 1000 × W;

Chl *b* (mg/g FW) = [(22.9 ×OD 645) − (4.68 × OD 663)] × V / 1000 × W;

Carotenoids (mg/g FW) = [(OD 480) − 0.114(OD 663) − 0.638(OD 645)] × 1000/2500;

where:

A - absorbance at different wavelengths;

V - solvent volume (mL);

W - weight of the tissue (g).

### Estimation of MDA, proline, and H_**2**_O_**2**_ content

To determine MDA content in fresh leaves, the method described by Cakmak and Horst (1991) was used. Fresh leaves (0.5 g) were homogenized in 0.1% trichloroacetic acid (TCA). The obtained homogenate was centrifuged at 10,000 × g for 10 min. Then, 1 mL of the supernatant was mixed with 0.5% thiobarbituric acid (TBA) solution prepared in 20% TCA. The mixture was incubated in a water bath for 30 min at 90 °C. The reaction was stopped on ice, and the samples were centrifuged at 10,000 × g for 5 min. The absorbance of the supernatant was measured spectrophotometrically at wavelengths of 532 and 600 nm. MDA content was calculated using the formula:

MDA (nmol g⁻¹ FW) = [(A532 – A600) × V × 1000/ϵ] × W;

where:

ϵ is the specific extinction coefficient (= 155 mM cm⁻¹);

V is the volume of crushing medium;

W is the weight of the tissue (g).

To determine proline content, the method developed by Bates et al. (1973) was used. Fresh leaves (0.5 g) were homogenized in 3% sulfosalicylic acid solution. Then, 2 mL of the obtained homogenate was mixed with 2 mL of acid ninhydrin and 2 mL of glacial acetic acid, and the mixture was incubated for 60 min at 95 °C. After incubation, the sample was cooled, 4 mL of toluene was added, and the sample was centrifuged. Absorbance was measured at a wavelength of 520 nm. Proline concentration was determined on the basis of a standard curve (0–50 µg mL⁻¹), and the value was calculated using the formula:

µmole proline/g⁻¹ FW = [(µg proline/mL×mL toluene) / 115.5 µg/µmole] / [(g sample) /5]

Hydrogen peroxide content in soybean leaves was determined according to Velikova et al. (2000). Fresh leaves (0.5 g) were homogenized in 0.1% trichloroacetic acid (TCA). The homogenate was centrifuged at 12,000 × g for 15 min. Then, 10 mM potassium phosphate buffer (pH 7.0) and 1 M KI were added to 0.5 mL of the supernatant. The absorbance of the supernatant was measured at 390 nm. H_2_O_2_ content was calculated by comparison with the H_2_O_2_ standard curve.

### Determination of antioxidant enzyme activity

Approximately 0.5 g of fresh material was homogenized in 5 mL of 50 mM chilled potassium phosphate buffer (pH 7.0) and 0.5 mM EDTA. The homogenate was centrifuged for 20 min at 12,000 × g at 4 °C. CAT enzyme activity in soybean leaves was determined according to the method of Chance and Maehly (1955). To 0.1 mL of plant extract, 5.9 mM H_2_O_2_ and 50 mM phosphate buffer (pH 7.0) were added. Absorbance was measured at 240 nm at 20-second intervals for 2 min. POD activity in leaves was determined by the method of Chance and Maehly (1955). A reaction mixture containing phosphate buffer (pH 7.0), H_2_O_2_ (40 mM), guaiacol (0.5%), and enzymatic extract (0.1 mL) was prepared. The increase in absorbance at 470 nm caused by guaiacol oxidation was monitored every 20 s for 2 min. APX activity was measured according to the method of Nakano and Asada (1981). To measure APX enzyme activity, a reaction mixture containing ascorbate (0.5 mM), phosphate buffer (pH 7.0), H_2_O_2_ (5.9 mM), and enzymatic extract was prepared. Then, absorbance was measured spectrophotometrically at 290 nm for 2 min at 20-second intervals.

### NIRS analyses

The collected soybean above-ground biomass (excluding pods) was dried at 70 °C and then ground into a fine powder. The content of total protein, crude fibre, crude ash, Neutral Detergent Fiber (NDF), Organic Matter Digestibility (OM Digestibility), Dry Matter Digestibility (DM Digestibility), and total soluble sugars (WSC) in the studied biomass was determined by the NIRS method using a NIRFlex N-500 near-infrared spectrometer (BuchiLabortechnik AG, Flawill, Switzerland) calibrated with pre-existing INGOT^®^ models specifically developed for dried forage (ISO 12099:^2017^).

### Soil physicochemical analysis and soil dehydrogenase activity

Soil samples were air-dried and sieved through a 2 mm mesh to determine pH, electrical conductivity (EC), total nitrogen (TN), available P, available K, available Ca, humus, and OC. The following methods were used to evaluate each parameter: pH was measured in H_2_O according to (PN-EN ISO 10390:2022-09 2022); TN was determined according to the Polish standard PN-EN 16169:2012; available P and K were measured according to Polish procedures PN-R-04023:1996, PN-R-04022:1996 + Az1:2002; available Ca was determined according to the method of Mehlich (1984). OC and humus were determined according to the Polish standard PN-EN ISO 10694:1995; EC was measured using a conductivity meter according to PN-ISO 11,265 + AC 1,1997.

Soil dehydrogenase activity was determined according to ISO 23753-1:2005. The procedure consisted of a 16-hour incubation of the soil sample at 25 °C in Tris buffer solution, with 1% TTC solution (2,3,5-triphenyltetrazolium chloride) as the substrate. After incubation, triphenyl tetrazolium formazan (TPF) was extracted using 99.5% acetone, and its concentration was measured spectrophotometrically at a wavelength of 485 nm. Enzymatic activity was expressed in units of µg TPF g⁻¹ h⁻¹. TPF concentration was determined on the basis of a standard curve (0–33.3 µg/mL).

### Statistical analyses

All experimental data from three independent annual pot experiments were analysed jointly using two-way analysis of variance (ANOVA) with salinity and inoculation as fixed factors at a significance level of *p* ≤ 0.05. Each treatment consisted of four biological replicates (pots) per year, resulting in a total of 12 replicates per treatment across the three years. Each year was treated as an independent temporal replicate of the same factorial experimental design and no pre-averaging of data across years was performed prior to analysis. Results are presented as means ± standard deviation (SD) for each treatment in tables, while figures show mean values with standard error (SE). When significant differences among treatments were detected, Tukey’s HSD post hoc test was used to separate means at *p* ≤ 0.05. All statistical analyses were performed using Statistica 12.0 (StatSoft Inc., Tulsa, OK, USA).

## Results

### Salt, pH, and temperature tolerance of strain KF23

**Fig. 2:**
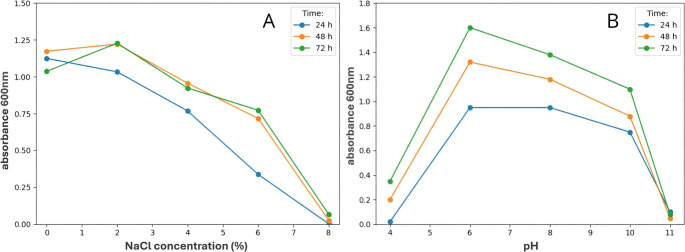
Effect of NaCl concentration (**A**) and pH (**B**) on the growth of KF23 during incubation (24, 48, and 72 h)

Within this study, a total of 32 bacterial strains were isolated, among which only one strain exhibited tolerance to salt stress. The results of KF23 tolerance to different NaCl concentrations (0–8%) and pH values evaluated after 24, 48, and 72 h of incubation are presented in Fig. 2A-B. KF23 showed clear growth up to 6% NaCl. The highest OD600 values occurred at 0–2% NaCl, with a maximum at 2% (1.03–1.26). Importantly, however, at 4 and 6% NaCl, high and stable OD_600_ values were still obtained after 48–72 h (0.718–0.956), indicating good tolerance to salinity stress. Above this level (8% NaCl), a rapid decline in growth occurred to values close to zero (0.003–0.068).

The optimal pH for KF23 growth ranged from 6 to 10. Although the highest growth was recorded at pH 6 after 72 h (1.616), the strain also showed high growth under alkaline conditions at pH 8–10 (0.737–1.364). In addition, isolate KF23 tolerated a wide temperature range from 6 to 42 °C.

### Molecular characterization and phylogenetic analysis of the bacterial isolate

**Fig. 3:**
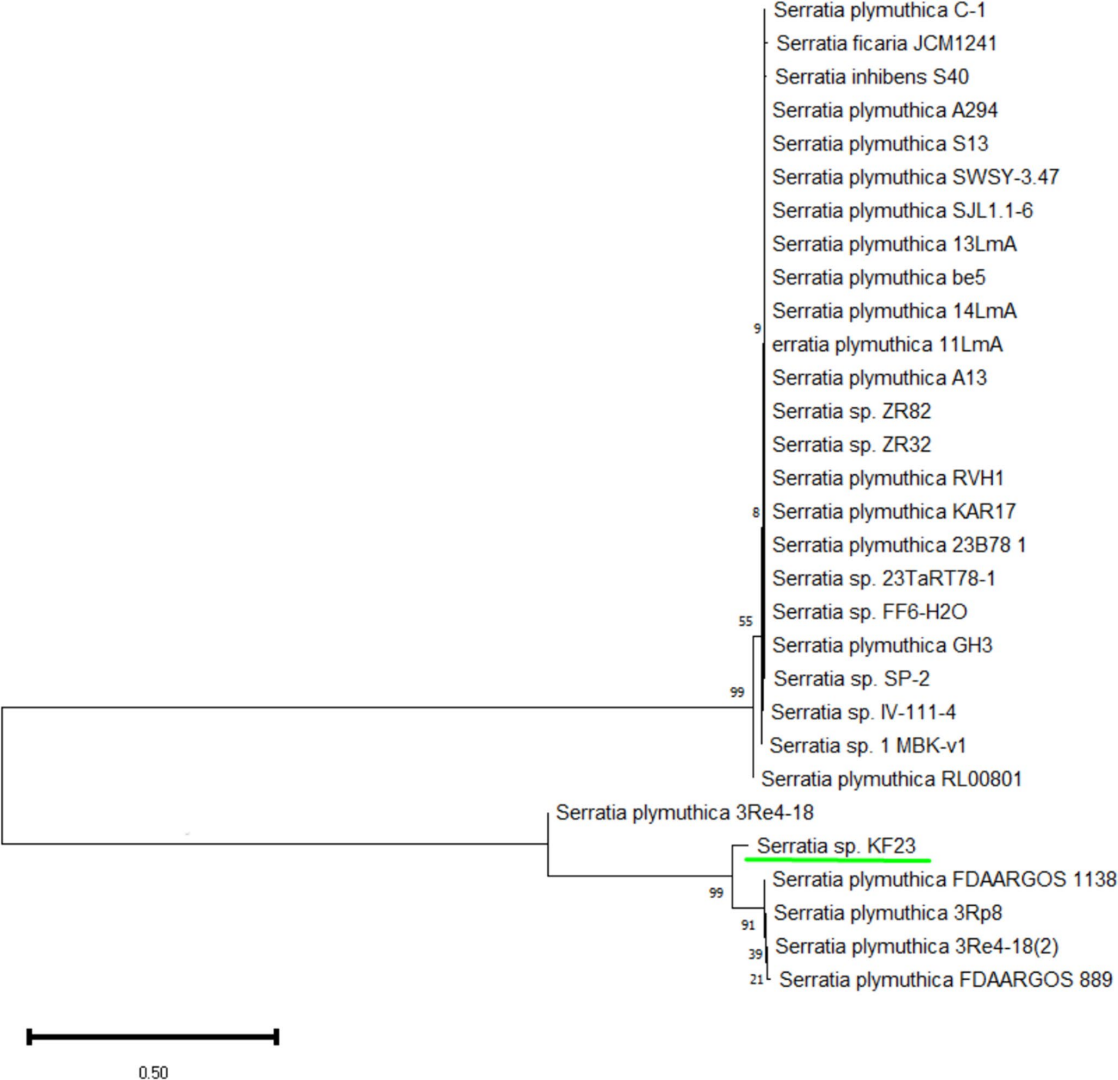
Phylogenetic tree of *Serratia* sp. KF23 was constructed using the Neighbor-Joining (NJ) method in MEGA 7 based on the 16 S rRNA gene sequence. Bootstrap values at the nodes represent percentages obtained from 1000 replicates. The scale bar (0.50) indicates the number of nucleotide substitutions per sequence site

The studied isolate KF23 was identified based on 16 S rRNA gene sequencing. Comparative analysis using the BLAST showed high sequence similarity of strain KF23 to sequences belonging to the genus *Serratia*. Phylogenetic analysis showed that strain KF23 clusters within the clade including *Serratia plymuthica* strains (Fig. 3). KF23 formed a common phylogenetic branch with reference isolates of *S. plymuthica*, including strains 3Re4-18, 3Rp8, and FDAARGOS 1138, with high bootstrap support values, which may confirm the close phylogenetic relationship of the studied isolate with representatives of this species. Due to the limited resolution of the 16 S rRNA marker within closely related species of the genus *Serratia*, the strain was designated as *Serratia* sp. KF23, with a clear phylogenetic relationship to *S. plymuthica*. The 16 S rRNA gene sequence of the studied bacterial strain was deposited in GenBank under accession number PZ122767.

### Biofilm- and EPS-forming potential of *Serratia* sp. KF23

**Fig. 4:**
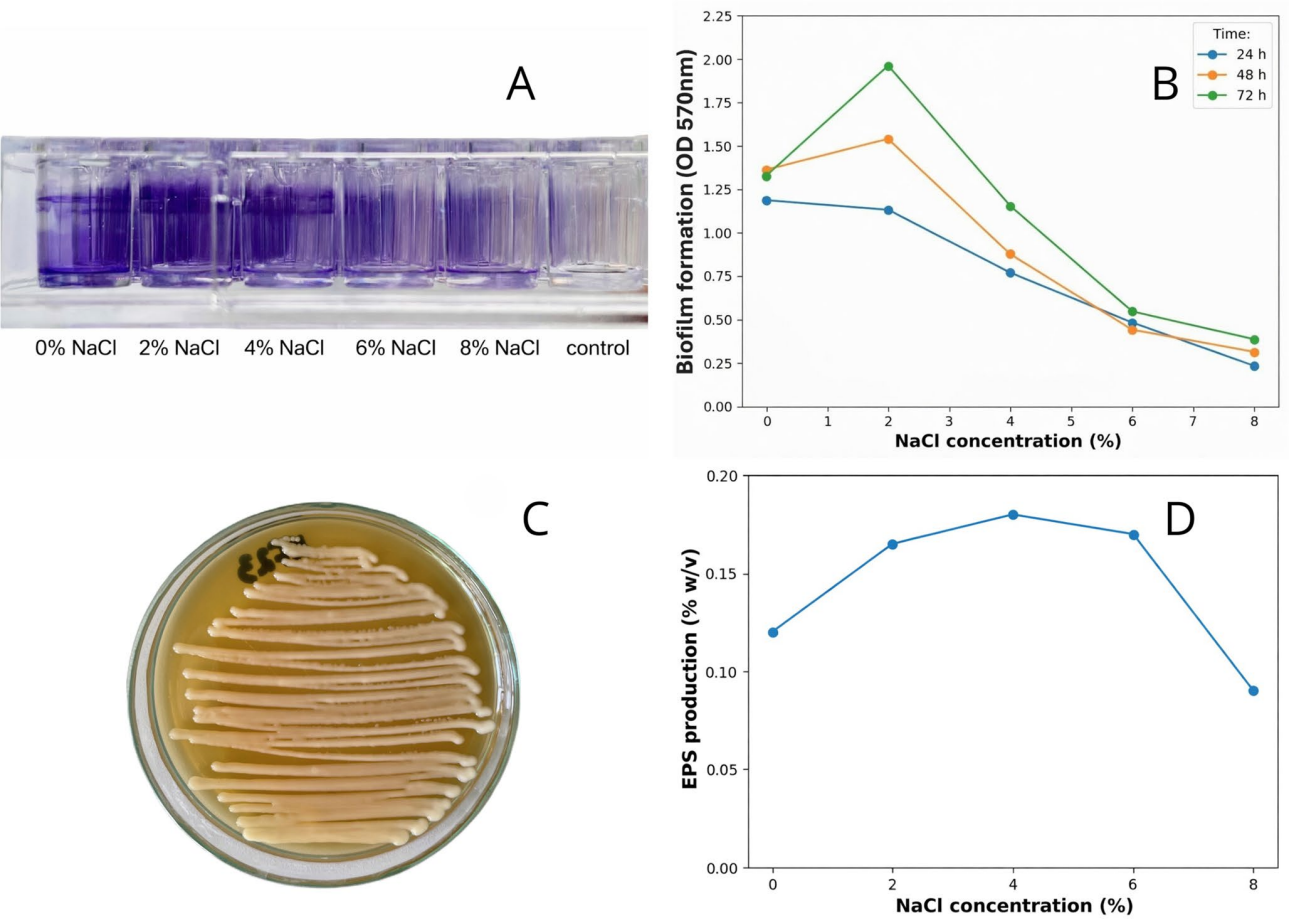
Biofilm formation by *Serratia* sp. KF23 (**A**) and the biofilm biomass (**B**) of the stained bacterial isolate measured at different time intervals (24, 48, and 72 h). The biomass of cells adhering to microtiter plates supplemented with five NaCl concentrations (0, 2, 4, 6, and 8%) was quantified by measuring absorbance at OD_570_ nm. Qualitative (**C**) and quantitative (**D**) EPS production by the strain *Serratia* sp. KF23

Biofilm formation by *Serratia* sp. KF23 was clearly dependent on NaCl concentration and incubation time (Fig. 4A-B). Extending the incubation time from 24 to 72 h increased biofilm formation across the entire tested range of NaCl concentrations. The highest biofilm intensity was observed under low salinity, with maximum growth at 2% NaCl, especially after 72 h of incubation (OD_570_ = 1.963). As the salt concentration increased to 4–6% NaCl, a gradual reduction in biofilm-forming ability occurred, but the biofilm biomass remained high and after 72 h reached OD_570_ = 1.156 at 4% NaCl and OD_570_= 0.547 at 6%. At the highest tested concentration (8% NaCl), biofilm formation was significantly limited at all time points.

Qualitative evaluation confirmed the ability of *Serratia* sp. KF23 to produce exopolysaccharides (EPS) (Fig. 4C). After 48 h of incubation, KF23 showed a distinct mucoid layer on the surface of the medium, indicating the synthesis of extracellular polysaccharides.

Quantitative analysis showed that EPS production by strain KF23 depended on the NaCl concentration in the culture medium (Fig. 4D). The lowest EPS production was recorded at the highest salinity level (8% NaCl), where it amounted to 0.09% (w/v). At 0% NaCl, the strain produced 0.12% (w/v) EPS. Increasing salinity to 2% NaCl caused an increase in EPS yield to 0.165% (w/v), whereas maximum production was recorded at 4% NaCl, reaching 0.18% (w/v). A slight decrease in EPS synthesis was observed at 6% NaCl (0.17% w/v), but production still remained relatively high compared with the control.

### Estimation of P and Zn solubilization, production of IAA, siderophore, NH_**3**_, and hydrolytic enzymes of *Serratia* sp. KF23

**Fig. 5:**
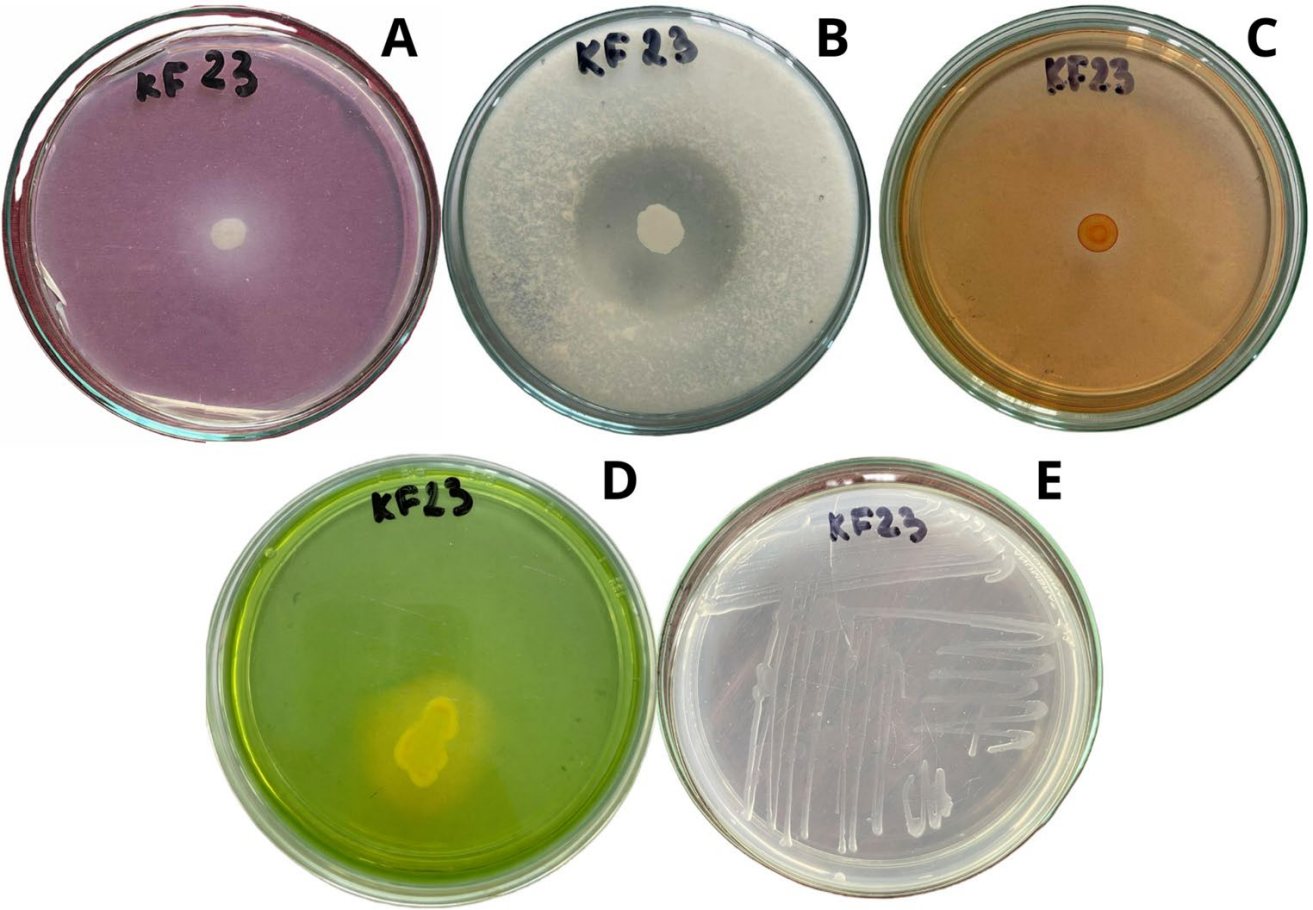
Assays for the *Serratia* sp. KF23 for solubilization ZnSO_4_ (**A**); proteolytic activity (**B**); β-1,3-glucanase production (**C**); siderophore production (**D**); nitrogen fixation (**E**)

IAA production, NH_3_ production, nitrogen fixation, hydrolytic enzymes, and P and Zn solubilization by strain *Serratia* sp. KF23 were determined under in vitro conditions (Table 1). The amount of IAA produced by isolate KF23 was 21.09 µg mL^− 1^. In addition, KF23 showed the ability to produce NH_3_, siderophores, fix atmospheric nitrogen, and solubilize ZnSO_4_ (Fig. 5A-E), whereas it did not show the ability to solubilize phosphorus and ZnO. Analysis of enzymatic activities showed the presence of proteolytic activity and β-1,3-glucanase, with no CMCase activity. Strain KF23 also exhibited catalase activity.

**Table 1:** Qualitative and quantitative characterization of PGP traits of *Serratia* sp. KF23

PGP traits	Serratia sp. KF23
P solubilization	—
ZnO solubilization	—
ZnSO_4_ solubilization	+
Siderophore production	+
IAA (µg mL^− 1^)	21.09
NH_3_	+
NH_3_ (µmole mL^− 1^)*	3.8
Nitrogen fixation	+
Proteolytic activity	+
β-1,3 - glucanase	+
CMCase	—

+ means positive activity and — means no activity

*NH_3_production attributes are equivalent to small:1–3 µmole mL^−1^,moderate:3–6 µmole mL^−1^,and large:  > 6 µmole mL^−1^

### Effect of *Serratia* sp. KF23 on soybean growth and biomass under salinity stress

**Fig. 6:**
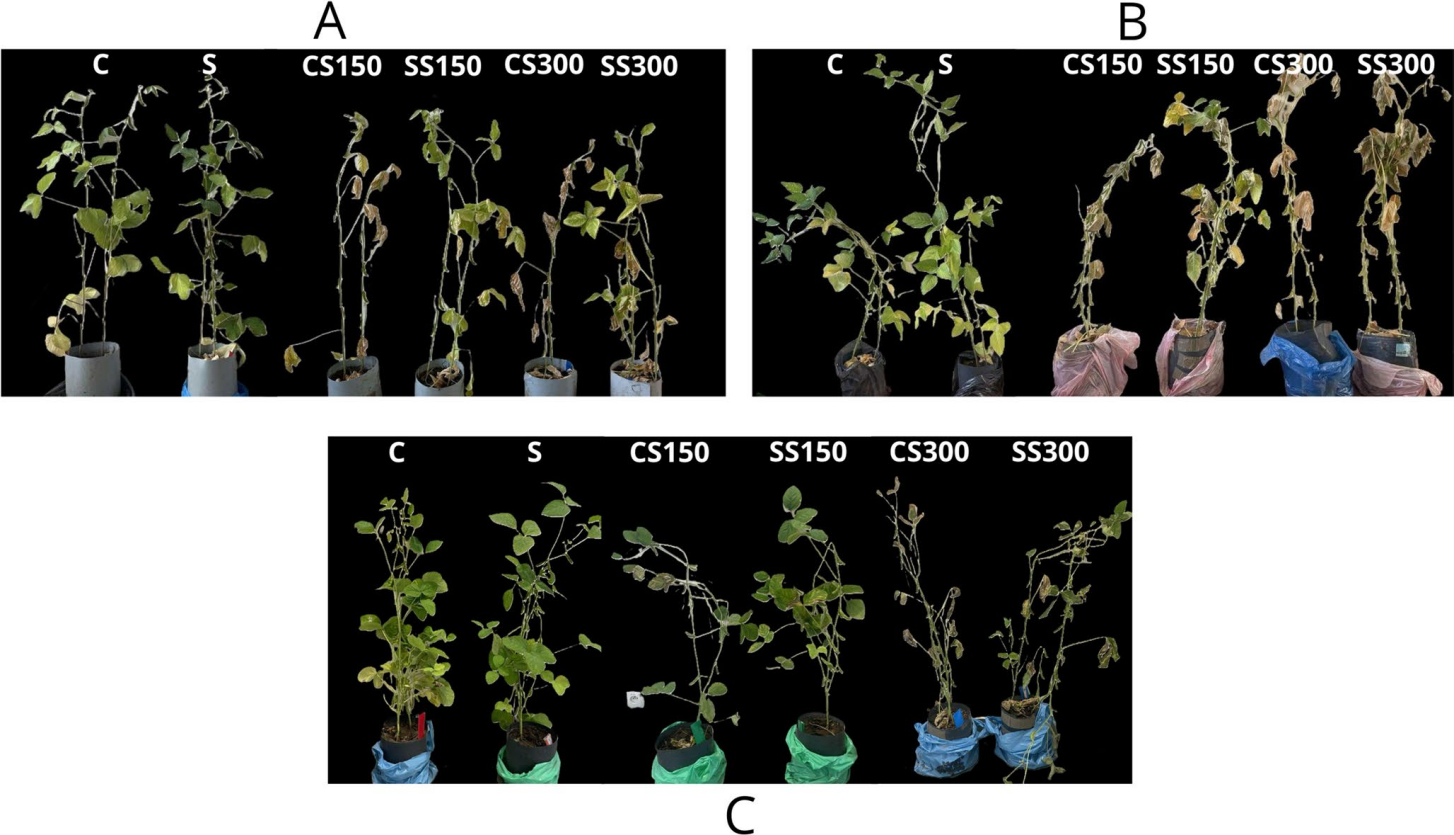
Soybean plants in a three-year pot experiment under different salinity levels (0, 150, and 300 mM NaCl): 2023 (**A**), 2024 (**B**), 2025 (**C**)

The averages from the three-year pot experiment (2023–2025) showed that salinity clearly limited soybean growth and biomass, whereas treatment with strain *Serratia* sp. KF23 alleviated the negative effects of salinity stress and improved most of the studied morphological parameters of plants (Fig. 6; Table 2). Under non-saline conditions, the application of *Serratia* sp. KF23 (S) statistically significantly improved shoot length, shoot fresh weight, and shoot dry weight compared with the control (C) by 3.3%, 12%, and 15.6%, respectively. In addition, an increase in fresh pod weight by 4.2% was recorded for inoculated seeds.

Under moderate salinity, inoculation with *Serratia* sp. KF23 (SS150) statistically significantly increased root length and shoot fresh weight compared with uninoculated plants (CS150) by 32% and 9.8%, respectively. The remaining studied parameters were statistically similar to the values obtained for control plants treated with 150 mM NaCl; nevertheless, the application of *Serratia* sp. KF23 showed an increasing tendency for all studied parameters. Under higher salinity (300 mM NaCl), the application of *Serratia* sp. KF23 (SS300) significantly alleviated the negative effect of salt on plants, which was reflected in statistically significantly higher values of shoot and root length, shoot fresh and dry weight, and fresh pod weight by 9.6%, 39.6%, 45.1%, 23.3%, and 36.9%, respectively, compared with uninoculated plants (CS300).

C - control (0 mM NaCl); S - *Serratia* sp. KF23 (0 mM NaCl); CS150- salinity (150 mM NaCl); SS150- salinity + *Serratia* sp. KF23 (150 mM NaCl); CS300- salinity (300 mM NaCl); SS300 - salinity + *Serratia* sp. KF23 (300 mM NaCl).

**Table 2:** Effect of *Serratia* sp. KF23 on shoot and root length, fresh and dry weight of shoots and roots, and fresh and dry weight of pods of soybean grown under salinity stress conditions in a three-year (mean for 2023–2025) pot experiment

Treatment	NaCl conc.	Shoot length (cm)	Root length (cm)	Shoot fresh weight (g/pot)	Shoot dry weight (g/pot)	Root fresh weight (g/pot)	Root dry weight (g/pot)	Pod fresh weight (g/pot)	Pod dry weight (g/pot)
C	0 mM	122.57 ± 14.1ab	48.26 ± 7.87ab	147.85 ± 45.5ab	28.00 ± 7.03ab	18.85 ± 4.29a	6.15 ± 1.75a	44.03 ± 22.6ab	12.32 ± 7.09a
*S*		126.65 ± 13.6a	58.17 ± 14.97ab	165.27 ± 39.9a	32.37 ± 6.92a	23.23 ± 11.48a	9.13 ± 4.93a	45.87 ± 17.0a	12.32 ± 5.35a
CS150	150 mM	114.81 ± 12.3ab	43.63 ± 11.92b	97.86 ± 34.4c	18.71 ± 6.14c	14.50 ± 7.05a	5.11 ± 2.26a	29.93 ± 18.9ab	8.4 ± 5.74a
SS150		124.02 ± 13.9ab	57.57 ± 8.35ab	107.46 ± 34.3bc	19.19 ± 6.41c	16.26 ± 8.34a	6.55 ± 2.70a	33.10 ± 19.0ab	9.20 ± 5.61a
CS300	300 mM	106.91 ± 24.9b	43.46 ± 14.54b	79.73 ± 33.7c	19.10 ± 7.77c	18.23 ± 14.45a	6.80 ± 6.07a	23.29 ± 15.4b	6.73 ± 5.53a
SS300		117.14 ± 14.1ab	60.67 ± 18.20a	115.66 ± 26.7bc	23.55 ± 6.93bc	21.96 ± 14.47a	9.69 ± 6.51a	31.88 ± 14.6ab	8.17 ± 4.66a

*C* control (0 mM NaCl), *S* Serratia sp. KF23 (0 mM NaCl), CS150- salinity (150 mM NaCl), SS150- salinity + Serratia sp. KF23 (150 mM NaCl), CS300- salinity (300 mM NaCl), SS300 - salinity +Serratia sp. KF23 (300 mM NaCl)

In each column, means followed by the same letter are not significantly different at p ≤ 0.05. Values are presented as mean ± SD (n = 12)

### Effects of salinity stress and bacterial seed treatment with *Serratia* sp. KF23 on plant chlorophyll content and carotenoids

The chl *a* content was the highest in plants not treated with NaCl (C) and significantly decreased with increasing salinity in uninoculated plants (Table 3). In contrast, in plants inoculated with strain KF23, the chl *a* level remained similar regardless of NaCl concentration, while at 150 mM NaCl (SS150) it was statistically significantly higher by 6.25% compared with treated uninoculated plants (CS150). Application of strain *Serratia* sp. KF23 caused a significant improvement in chl b content in plants not treated (S) with NaCl and treated with 300 mM NaCl (SS300) by 62.2% and 54.9%, respectively, compared with uninoculated plants. In the variant with plants treated with 150 mM NaCl, despite the lack of a statistically significant difference, strain *Serratia* KF23 increased chl *b* content by 13.5% relative to CS150 plants. The content of total chl and carotenoids did not show significant differences among variants. With increasing NaCl concentration, an upward trend in carotenoid content was observed in both uninoculated plants and those inoculated with *Serratia* sp. KF23. The highest values were recorded in the SS300 variant; however, differences among treatments were not statistically significant.

**Table 3:** Effect of *Serratia* sp. KF23 on the content of chl *a*, chl *b*, total chl, and carotenoids in soybean leaves grown under salinity stress conditions in a three-year (mean for 2023–2025) pot experiment

Treatment	NaCl conc.	Chl a (mg/g FW)	Chl b (mg/g FW)	Total Chl (mg/g FW)	Carotenoids (mg/g FW)
C	0 mM	0.58 ± 0.02a	0.45 ± 0.17b	1.03 ± 0.19a	1.09 ± 0.34a
*S*		0.57 ± 0.01ab	0.73 ± 0.11ab	1.30 ± 0,10a	1.49 ± 0.25a
CS150	150 mM	0.48 ± 0.14b	0.52 ± 0.32ab	1.01 ± 0,45a	1.33 ± 0.65a
SS150		0.51 ± 0.09ab	0.59 ± 0.33ab	1.10 ± 0,42a	1.34 ± 0.62a
CS300	300 mM	0.52 ± 0.07ab	0.51 ± 0.30ab	1.03 ± 0.36a	1.31 ± 0.52a
SS300		0.55 ± 0.04ab	0.79 ± 0.32a	1.34 ± 0.34a	1.61 ± 0.58a

*C* control (0 mM NaCl), *S* Serratia  sp. KF23 (0 mM NaCl), CS150- salinity (150 mM NaCl), SS150- salinity + Serratia sp. KF23 (150 mM NaCl), CS300- salinity (300 mM NaCl), SS300 - salinity +Serratia sp. KF23 (300 mM NaCl)

In each column, means followed by the same letter are not significantly different at p ≤ 0.05. Values are presented as mean ± SD (n = 12)

### Effects of bacterial seed treatment with *Serratia* sp. KF23 on MDA, proline, and H_**2**_O_**2**_ under salinity stress conditions

**Fig. 7:**
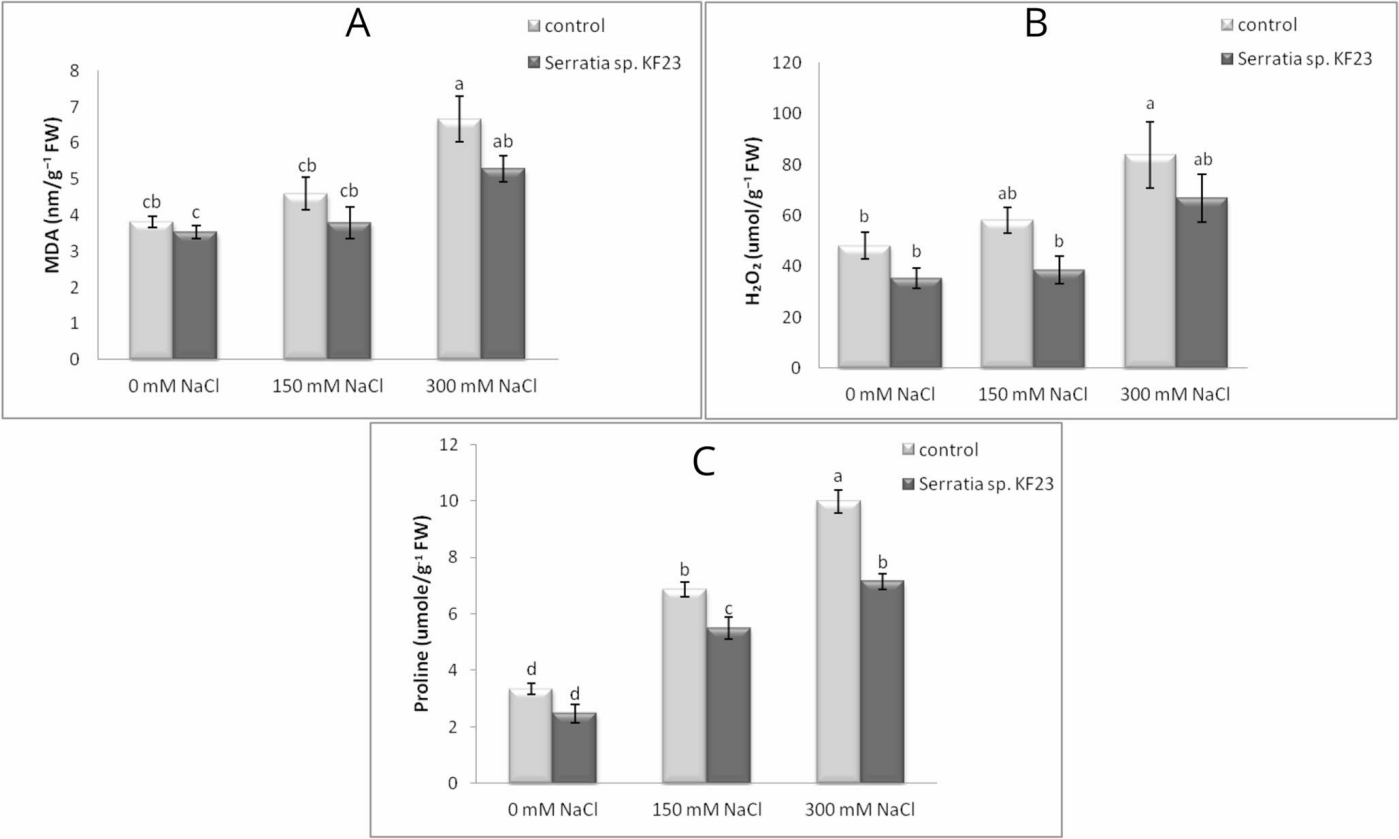
Effect of *Serratia* sp. KF23 on the content of MDA (**A**), H_2_O_2_ (**B**), and proline (**C**) in soybean leaves grown under salinity stress conditions in a three-year (mean for 2023–2025) pot experiment. Error bars represent standard error (SE) of the mean (*n* = 12). Different letters above the bars indicate significant differences at *p* ≤ 0.05

A statistically significant reduction in MDA level was observed in plants inoculated with KF23 (S) and treated with 300 mM NaCl (SS300) by 7.1% and 20.7%, respectively, compared with control plants (C and CS300). Plants treated with 150 mM NaCl in the average of the 3-year pot experiment showed differences between variants and a reduction in MDA level by 17.4% in plants inoculated with KF23 (SS150) compared with CS150; however, these differences were not statistically significant (Fig. 7A). Inoculation of soybean plants with *Serratia* sp. KF23 also did not cause a statistically significant change in H_2_O_2_ content compared with uninoculated plants under non-saline conditions (0 mM NaCl) (Fig. 7B). However, the H_2_O_2_ level was significantly reduced under moderate salinity (150 mM NaCl; decrease by 33.5%) and high salinity (300 mM NaCl; decrease by 20.3%) compared with control plants (CS150 and CS300). The results indicate that increasing salinity caused increased proline accumulation, whereas plants treated with *Serratia* sp. KF23 showed a lower proline level compared with the control (Fig. 7C). However, under non-saline stress conditions (0 mM NaCl), inoculation with *Serratia* sp. KF23 caused a decrease in proline level by 26.0% compared with control plants (C). At concentrations of 150 mM NaCl and 300 mM NaCl, the proline content in inoculated plants (SS150) was 20.1% and 28.5%, respectively, lower compared with control plants (CS150, CS300).

### Effects of *Serratia* sp. KF23 on antioxidant enzymes under salinity stress conditions

**Fig. 8:**
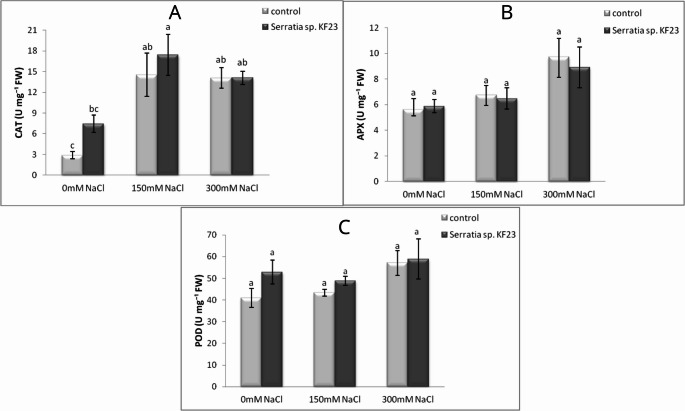
Effect of *Serratia sp.* KF23 on the content of CAT (**A**), APX (**B**), and POD (**C**) in soybean leaves grown under salinity stress conditions in a three-year (mean for 2023–2025) pot experiment. Error bars represent standard error (SE) of the mean (*n* = 12). Different letters above the bars indicate significant differences at *p* ≤ 0.05

CAT activity was the lowest in the leaves of control plants (C) without NaCl and clearly increased under salinity (Fig. 8A). The application of *Serratia* sp. KF23 statistically significantly increased CAT activity under non-saline conditions (S) by 157.6% relative to C and at 150 mM NaCl (SS150) by 20.0% relative to CS150. In contrast, at 300 mM NaCl, CAT values were practically identical in both variants.

APX activity showed an upward trend with increasing NaCl concentration, reaching the highest values at 300 mM NaCl (Fig. 8B). However, no statistically significant differences were recorded among variants. In the case of POD, an increase in enzyme activity was observed with increasing salinity, especially in the control (Fig. 8C). KF23 inoculation statistically non-significantly increased POD at all salinity levels, most strongly under non-NaCl conditions (S) by 29.3% relative to control plants (C) and at 150 mM NaCl (SS150) by 12.4% relative to CS150. At 300 mM NaCl, the effect of KF23 was slight.

### Physicochemical properties of soil

Inoculation with KF23 caused several statistically significant changes in the physicochemical properties of the soil (Table 4). EC was clearly determined by the NaCl salinity level. In the control variant (0 mM), EC was 0.81–0.86 dS m^-1^, whereas under moderate salinity stress (150 mM NaCl) it significantly increased to 3.38–3.42 dS m^-1^. The highest EC values were recorded in soil treated with 300 mM NaCl, where EC ranged from 5.61 to 5.76 dS m^-1^. The soil remained slightly alkaline (7.1–7.25) throughout the pot experiment period (2023–2025), and regardless of salinity and inoculation, no significant differences in pH levels were observed. In the SS300 variant, TN content showed a statistically significant response to KF23 inoculation and the mean over 3 years was higher (3008.8 mg kg⁻¹) compared with the corresponding uninoculated variant CS300 (2700.8 mg kg⁻¹) by 11.4%. The contents of available forms of Ca, K, and P did not differ significantly among experimental variants, similarly to the content of OC and humus.

**Table 4:** Physicochemical properties of soil in the pot experiment (mean for 2023–2025) as affected by NaCl salinity (0, 150, and 300 mM) and *Serratia* sp. KF23 application

Treatment	NaCl conc.	pH (H_2_O)	EC (dS m^-1^)	TN (mg/kg⁻¹)	Ca available (mg/kg⁻¹ DW)	K available (mg/kg⁻¹ DW)	*P* available (mg/kg⁻¹ DW)	OC (%)	Humus (% m/m)
C	0 mM	7.25 ± 0.25a	0.86 ± 0.26a	2772.43 ± 282.31ab	2825.92 ± 459.92a	535.61 ± 231.33a	257.25 ± 51.92a	2.51 ± 0.36a	4.36 ± 0.47a
*S*		7.24 ± 0.21a	0.81 ± 0.32a	2816.10 ± 135.35ab	2766.96 ± 388.74a	543.12 ± 221.57a	261.82 ± 40.55a	2.41 ± 0.35a	4.00 ± 0.52a
CS150	150 mM	7.10 ± 0.20a	3.42 ± 0.62b	2607.80 ± 87.09b	2572.84 ± 197.68a	498.47 ± 181.06a	253.92 ± 33.58a	2.26 ± 0.17a	4.15 ± 0.47a
SS150		7.17 ± 0.17a	3.38 ± 0.60b	2645.78 ± 123.83b	2637.69 ± 219.49a	489.91 ± 146.54a	245.46 ± 13.91a	2.22 ± 0.31a	3.98 ± 0.57a
CS300	300mM	7.16 ± 0.13a	5.76 ± 0.87c	2700.78 ± 334.03b	2701.99 ± 234.77a	551.60 ± 173.12a	255.97 ± 28.86a	2.49 ± 0.38a	4.15 ± 0.59a
SS300		7.16 ± 0.12a	5.61 ± 0.95c	3008.82 ± 332.57a	2880.14 ± 293.18a	580.60 ± 220.90a	261.78 ± 33.22a	2.33 ± 0.28a	4.07 ± 0.44a

*C* control (0 mM NaCl), *S* *Serratia* sp. KF23 (0 mM NaCl), CS150- salinity (150 mM NaCl), SS150- salinity + *Serratia* sp. KF23 (150 mM NaCl), CS300- salinity (300 mM NaCl), SS300 - salinity + *Serratia* sp. KF23 (300 mM NaCl).

In each column, means followed by the same letter are not significantly different at p ≤ 0.05. Values are presented as mean ± SD (n = 12)

### Soil dehydrogenase activity

**Fig. 9:**
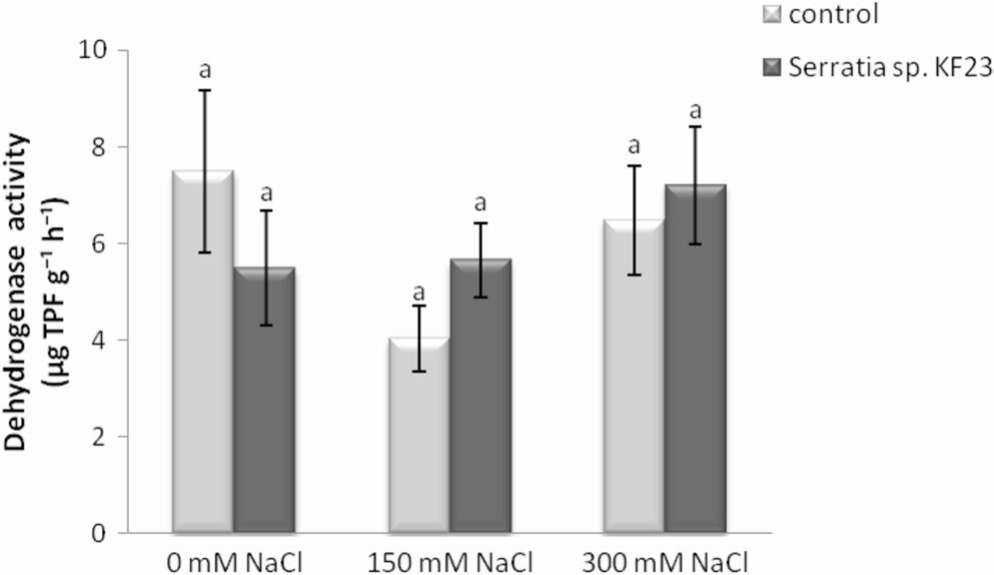
Effect of *Serratia* sp. KF23 on soil dehydrogenase activity under salinity stress in a three-year pot experiment (mean for 2023–2025). Error bars represent standard error (SE) of the mean (*n* = 12). Different letters above the bars indicate significant differences at *p* ≤ 0.05

The three-year average soil dehydrogenase activity did not differ statistically significantly between soil with control plants (C) and soil with plants inoculated with strain *Serratia* sp. KF23 (S) in all analyzed salinity variants (0, 150, and 300 mM NaCl) (Fig. 9). Despite the lack of statistically significant differences, under moderate and high salinity conditions (150 and 300 mM NaCl) a tendency toward higher dehydrogenase activity was observed in the soil with plants inoculated with strain KF23 compared with soil with uninoculated plants by 40.3% and 11.1%, respectively. In contrast, under non-saline conditions (0 mM NaCl), dehydrogenase activity values in uninoculated plants were higher than after application of strain KF23 (decrease by 26.7%). Interestingly, soil dehydrogenase values in soil inoculated with KF23, regardless of NaCl level, remained at a similar level (5.49 µg TPF g⁻¹ h⁻¹, 5.65 µg TPF g⁻¹ h⁻¹, and 7.2 µg TPF g⁻¹ h⁻¹), whereas in variants C, CS150, and CS300 dehydrogenase values were clearly more variable (7.49 µg TPF g⁻¹ h⁻¹, 4.03 µg TPF g⁻¹ h⁻¹, and 6.48 µg TPF g⁻¹ h⁻¹).

### Effect of *Serratia* sp. KF23 on chemical composition and digestibility in soybean leaves under salinity stress conditions

The lowest protein content was recorded in control plants C (Table 5). KF23 inoculation increased protein content in variants without NaCl and under the highest salinity conditions (S and SS300) by 35.8% and 22.5%, respectively, compared with control plants (C and CS300). Crude fibre content did not differ significantly among variants. Ash was significantly higher in saline variants compared with variants without salt. This means that salinity was the main factor increasing the content of mineral components, and inoculation did not significantly differentiate ash levels within salinity. NDF (%) was highest in variants C and SS150 (43.96 and 44.08, respectively) and lowest in SS300 (39.95). The remaining variants had intermediate values. This indicates that under the highest salinity, KF23 inoculation was associated with a decrease in the NDF fraction.

OM and DM digestibility showed significant differences. The lowest values were recorded under 150 mM NaCl conditions (CS150 and SS150; OM: 42.66–41.46% and DM: 42.26–41.49%). In the control (C) and in S as well as CS300, digestibility was intermediate. The highest digestibility was obtained in SS300 (OM 50.41% and DM 49.79%, significantly higher than in the 150 mM variants. WSC was highest in the non-saline variants (C and S; 15.47 and 14.86) and significantly lower in all saline variants (CS150-SS300; about 6.11–7.14). Thus, salinity strongly reduced the accumulation of soluble sugars, and KF23 inoculation did not restore them to the control level.

**Table 5:** Effect of *Serratia* sp. KF23 on the chemical composition and digestibility of soybean above-ground biomass (excluding pods) under different salinity levels (0, 150, and 300 mM NaCl) in a three-year pot experiment (mean for 2023–2025)

Treatment	NaCl conc.	Total protein (% DM)	Crude fibre (% DM)	Crude ash (% DM)	NDF (% DM)	OM Digestibility (%)	DM Digestibility (%)	WSC (% DM)
C	0 mM	5.87 ± 1.31d	29.49 ± 4.04a	8.25 ± 0.91b	43.96 ± 3.42a	45.51 ± 7.00ab	43.83 ± 5.46ab	15.47 ± 5.99a
*S*		7.97 ± 1,86 cd	28,68 ± 3.39a	8.52 ± 1.02b	43.03 ± 2.75ab	48.05 ± 7.06ab	46.73 ± 5.39ab	14.86 ± 5.51a
CS150	150mM	9.40 ± 2.28bc	28.17 ± 2.53a	14.28 ± 2.91a	42.40 ± 2.14ab	42.66 ± 6.47b	42.26 ± 6.47b	6.87 ± 4.25b
SS150		9.58 ± 1.77bc	29.90 ± 1.88a	13.50 ± 2.16a	44.08 ± 1.94a	41.46 ± 4.89b	41.49 ± 5.44b	6,53 ± 2.28b
CS300	300mM	10.54 ± 1.60b	29.10 ± 3.99a	13.61 ± 1.56a	42.93 ± 4.19ab	43.52 ± 7.45ab	43.01 ± 7.21ab	6,11 ± 1.70b
SS300		12.91 ± 2.00a	26.35 ± 2.39a	13.99 ± 1.50a	39.95 ± 2.77b	50.41 ± 4.67a	49.79 ± 5.23a	7.14 ± 1.47b

*C* control (0 mM NaCl), *S* *Serratia* sp. KF23 (0 mM NaCl), CS150- salinity (150 mM NaCl), SS150- salinity + *Serratia* sp. KF23 (150 mM NaCl), CS300- salinity (300 mM NaCl), SS300 - salinity + *Serratia* sp. KF23 (300 mM NaCl).

In each column, means followed by the same letter are not significantly different at p ≤ 0.05.  Values are presented as mean ± SD (n = 12)

## Discussion

Saline soils constitute one of the most serious threats to global food security, limiting the yield of key crop plants such as soybean (*Glycine max* L. Merr.). We isolated and characterized the strain *Serratia* sp. KF23, which not only exhibits moderate resistance to salt stress, but also supports soybean development under salinity conditions.

In the present study, KF23 showed intensive growth up to 6% NaCl, especially after 48 and 72 h of incubation; therefore, the studied strain can be considered moderately halotolerant and capable of functioning and adapting under conditions where most microorganisms lose viability (Mainka et al. 2021). Importantly, the literature review devoted to *Serratia* sp. emphasizes that within this genus, strains tolerating salinity of about 5.8–10% NaCl have been described, while at the same time exhibiting PGP traits (Kulkova et al. 2024). In addition, strain KF23 tolerated a wide range of pH (6–10) and temperature (6–42 °C), suggesting that the isolate possesses high phenotypic plasticity, which is essential for survival in different types of cultivation substrates and variable climatic conditions (Roriz et al. 2023; Stegelmeier et al. 2022).

The 16 S rRNA analysis performed indicated the affiliation of KF23 to the genus *Serratia* and a close relationship with the species *S. plymuthica*. The genus *Serratia* is increasingly recognized as a group of effective PGPB, and strains of *S. plymuthica* are well known for their biocontrol potential (Benhamou et al. 2000; Kurze et al. 2001; Ovadis et al. 2004; Vleesschauwer and Höfte 2007; Adam et al. 2016). Moreover, a product named RhizoStar (e-nema GmbH, Raisdorf, Germany) was developed based on *S. plymuthica* HRO-C48, which confirms the practical potential of representatives of this species in agricultural applications (Müller and Berg 2008). Nevertheless, only a few studies have shown that *S. plymuthica* strains have the ability to alleviate abiotic stress (Nordstedt and Jones 2021; Egamberdieva et al. 2011).

The results of the present study showed that KF23 synthesized IAA, produced siderophores, NH_3_, and exhibited the ability to fix nitrogen and solubilize ZnSO_4_. Additionally, it showed proteolytic activity and β-1,3-glucanase activity. Moreover, the strain formed a moderate biofilm at 0–6% NaCl, which increased over time up to 72 h, as well as EPS at 0–8% NaCl.

The biometric parameters showed that in the three-year pot experiment (2023–2025), the application of the strain *Serratia* sp. KF23 significantly increased soybean biometric parameters both under saline and non-saline (normal) conditions. At 300 mM NaCl (SS300), statistically significant increases were obtained in shoot length (+ 9.6%), root length (+ 39.6%), shoot fresh weight (+ 45.1%), shoot dry weight (+ 23.3%), and fresh pod weight (+ 36.9%) relative to CS300. At 150 mM NaCl (SS150), the key effects were the increase in root length (+ 32%) and the increase in shoot fresh weight (+ 9.8%). This effect may result from several mechanisms of action of strain KF23. The IAA produced by KF23 directly stimulated plant growth and favorably modified root system architecture under both normal conditions and salinity stress (Patel et al. 2024). Similar effects have been reported for other IAA-producing *Serratia* strains, including *S. marcescens* CDP-13 and *Serratia* sp. AP9 and AP12 in wheat exposed to 75–225 mM NaCl (Singh and Jha 2016a; Tugbaeva et al. 2026), as well as *S. marcescens* VIRS2 in rice cultivated under saline conditions (100 mM NaCl) (Ho et al. 2025). Moreover, an important protective mechanism of strain KF23 is the production of biofilm and exopolysaccharides (EPS). Biofilm- and EPS-producing bacteria form a protective layer around the roots, which enhances water retention, chelates and immobilizes toxic Na⁺ ions, stabilizes plant cell plasma membranes, and reduces electrolyte leakage and oxidative damage induced by salinity stress (Cakmak et al. 1989; Bhattacharyya and Pati 2000; Qurashi and Sabri 2012). Our results for KF23 are consistent with previous observations (Han and Lee 2005a, b; Ho et al. 2025; Maxton et al. 2018). In the study by Han and Lee (2005a, b), greenhouse experiments evaluated the effect of *S. proteamaculans* ATCC 35,475 (SP) on soybean grown in saline soils with electrical conductivity (EC) values of 1.5 and 5.0 dS m⁻¹, demonstrating that inoculation increased both plant and root biomass compared with the control, which was associated with EPS production. In another study, the biofilm- and EPS-producing strain *S. marcescens* VIRS2, capable of growth at 8% NaCl, enhanced the growth and salinity tolerance of rice under 100 mM NaCl conditions (Ho et al. 2025).

At the physiological level, long-term salinity stress usually leads to premature senescence of mature leaves and degradation of photosynthetic pigments (Mokrani et al. 2020). In our experiment, the application of KF23 limited this effect, which is visible in Fig. 6. In addition, the content of chl *a* was higher in SS150 plants by 6.25% than in CS150, and chl *b* increased particularly under severe salinity (SS300: +54.9% relative to CS300). This effect may have resulted from siderophore production, N_2_ fixation, and NH_3_ production, which could increase Fe bioavailability and support plant N supply, thereby promoting chlorophyll biosynthesis and limiting secondary oxidative stress (Kumar et al. 2020; Zayed et al. 2023).

The obtained results indicate that inoculation with *Serratia* sp. KF23 significantly reduced the levels of key oxidative stress markers (H_2_O_2_ and MDA). Interestingly, under non-saline conditions and moderate salinity (150 mM NaCl), KF23 inoculation significantly increased CAT activity compared to the controls, whereas at 300 mM NaCl, CAT activity was comparable between treatments. A similar concentration-dependent pattern of CAT modulation has been reported in canola (*Brassica napus* L.) inoculated with *Bacillus subtilis* SB (Tanur Erkoyuncu et al. 2026). At the same time, no significant changes were observed in APX and POD activities in the present study. This pattern may indicate a selective rather than global activation of the antioxidant system. A comparable selective induction of CAT, accompanied by no increase or even a decrease in the activity of other antioxidant enzymes (APX, guaiacol peroxidase- GPX, and glutathione reductase-GR), has been reported following inoculation with *Azospirillum brasilense* and *Azotobacter chroococcum* in coriander (*Coriandrum sativum* L.), as well as after treatment of lettuce (*Lactuca sativa* L.) with *Serratia* sp. ATCC 35,475 under salinity stress (Rabiei et al. 2020; Han and Lee 2005a, b). Importantly, in our three-year pot experiment, proline concentration in plants inoculated with KF23 was 20.1–28.5% lower compared to control plants. Although proline plays a well-documented role in osmotic regulation, it is also widely recognized as a stress marker (Zaki et al. 2025; Vives-Peris et al. 2018). Therefore, the reduced accumulation of proline suggests that inoculated plants were less exposed to salinity stress. Our findings are consistent with previous reports (Singh and Jha 2016b; Isfahani et al. 2018), which demonstrated that inoculation with *Serratia* sp. SL-12 and *Citrobacter freundii* KX553903 led to decreased proline levels in wheat and tomato leaves under salinity conditions, while simultaneously alleviating the negative effects of salt stress.

The three-year mean of the pot experiment showed that inoculation with KF23 under 300 mM NaCl conditions (SS300) resulted in a significant increase in total nitrogen (TN) content in the soil by 11.4% compared to the control (CS300). Our results are consistent with the results of Wang and Xu (2025), who recorded an improvement in total nitrogen and hydrolyzable nitrogen in soils contaminated with heavy metals after the application of *Serratia* sp. C20. Available studies indicate that PGPB strains with proteolytic activity and nitrogen-fixing capacity can affect nitrogen cycling in soil through protein hydrolysis and nitrogenase production, increasing the nitrogen pool in soil (Chen et al. 2023; Peng et al. 2023). In the present study, no statistically significant differences were observed in dehydrogenase activity between the control treatments and those inoculated with *Serratia* sp. KF23, which may indicate a lack of a pronounced effect of this strain on the overall microbial activity of the soil under the tested conditions.

In the above-ground soybean biomass (without pods) under 300 mM NaCl (SS300), the application of KF23 increased crude protein content by 22.5%, reduced the NDF fraction by 6.9%, and increased the digestibility of organic matter and dry matter (OM to 50.41% and DM to 49.79%) relative to control plants (CS300).Similar effects of PGPB inoculation on forage quality have been reported in other legumes (*Vigna unguiculata* (L.) Walp), where bacterial strains, including *S. marcescens* PGB15, increased crude protein content while reducing fiber fractions such as NDF and ADF, resulting in improved feed value of plant biomass (Severoğlu 2025). The improvement in soybean biomass composition observed in this study may be related to the increased N availability in soil following inoculation with *Serratia* sp. KF23. It may promote nitrogen uptake and stimulate protein synthesis in plant tissues. At the same time, improved plant nutritional status and growth stimulation induced by *Serratia* sp. may alter carbon allocation within plant biomass, leading to relatively lower accumulation of structural carbohydrates, which constitute the major components of the NDF fraction.

## Conclusions

This study provides novel evidence from a three-year pot experiment and, to the best of our knowledge, represents the first report demonstrating the consistent effects of *Serratia* sp. KF23 on soybean performance under salinity stress. It contributes to the limited body of knowledge on the long-term reproducibility of PGPB-mediated responses.

From a practical perspective, the obtained results suggest that inoculation with *Serratia* sp. KF23 may serve as an effective and environmentally friendly strategy to improve soybean performance and biomass quality in salt-affected soils, contributing to more sustainable agricultural practices in areas exposed to salinization. However, these implications should be interpreted with caution, as the results were obtained under controlled pot conditions and may not fully reflect the complexity of field environments. Therefore, the current study should not be viewed as a direct recommendation for agricultural application, and field-based validation under diverse environmental conditions, including varying salinity levels, soil types, and management practices, is essential before considering practical implementation.

It is also worth noting that, although *Serratia* spp. are widely reported as biocontrol agents and PGPB, this genus also includes opportunistic pathogens such as *S. marcescens* and *S. liquefaciens*. *S. plymuthica* is considered a very rare human and fish pathogen, reported only in a limited number of case reports and case series, and its pathogenic potential remains unclear and debated (Mahlen 2011; Singh and Pandey 2025; Nuñez 1990). Therefore, the application of KF23 requires careful biosafety assessment, including evaluation of genomic features related to virulence and antibiotic resistance.

Furthermore, future research should focus on the potential impact of *Serratia* sp. KF23 on native microbial communities associated with soybean plants and soil under salinity conditions, including root nodule formation, nitrogen fixation efficiency, root colonization, and ion homeostasis in soybean cultivation. Such studies will be critical to determine whether the promising effects observed under controlled conditions can be translated into reliable and safe applications in sustainable agriculture.

## Supplementary Information

Below is the link to the electronic supplementary material.

Supplementary Material 1

Supplementary Material 2

## Data Availability

No datasets were generated or analysed during the current study.
