# Supplementary material for: Potential of Serratia sp. KF23 in stimulating soybean growth and alleviating the effects of salinity stress in a three-year pot experiment
Source: World J Microbiol Biotechnol. 2026 May 30;42(6):318. doi: 10.1007/s11274-026-05055-0 (PMC13222251; doi:10.1007/s11274-026-05055-0)
Supplement: Supplementary file 1 — Supplementary Material 1 [file 11274_2026_5055_MOESM1_ESM.pdf]

Supplementary Table 1

Mean temperature and mean maximum and minimum temperatures in the polytunnel during the soybean growing period in 2023–2025

	2023			2024			2025		
	May	June	July	May	June	July	May	June	July
T	14.8	22.8	24.6	24.7	24	23.9	16.8	22.5	24.9
TM	20.8	28.8	30.9	30.3	30.5	30.3	22.3	28.6	29.4
Tm	9.6	16	18.1	16.9	17.5	17.4	11	15.9	16.3

T – mean temperature (°C); TM – mean maximum temperature (°C); Tm – mean minimum temperature (°C).
