# Supplementary material for: Potential of Serratia sp. KF23 in stimulating soybean growth and alleviating the effects of salinity stress in a three-year pot experiment
Source: World J Microbiol Biotechnol. 2026 May 30;42(6):318. doi: 10.1007/s11274-026-05055-0 (PMC13222251; doi:10.1007/s11274-026-05055-0)
Supplement: Supplementary file 2 — Supplementary Material 2 [file 11274_2026_5055_MOESM2_ESM.pdf]

Supplementary Information

Table 1 Effect of *Serratia* sp. KF23 on shoot and root length, fresh and dry weight of shoots and roots, and pod fresh and dry weight of soybean grown under salinity stress conditions in a three-year (2023, 2024, and 2025) pot experiment

**Treatment**	**NaCl conc. (mM)**	**Shoot length (cm)**	**Root length (cm)**	**Shoot fresh weight (g/pot)**	**Shoot dry weight (g/pot)**	**Root fresh weight (g/pot)**	**Root dry weight (g/pot)**	**Pod fresh weight (g/pot)**	**Pod dry weight (g/pot)**
		1st year of pot experiment (2023)							
C	0	109.56a	48.75ab	87.7b	23.83b	18.38a	6.87a	16.45abc	3.90ab
S		117.33a	46.67ab	115.69a	31.95a	14.88ab	6.19ab	26.14a	6.10a
CS150	150	102.69a	38.19b	53.32cd	13.26c	7.60d	3.93c	8.72c	1.99b
SS150		108.63a	63.00a	64.24c	14.73c	9.20cd	4.59bc	11.96bc	3.09b
CS300	300	79.88b	36.37b	39.34d	12.59c	8.74cd	4.03c	5.76c	1.30b
SS300		102.82a	48.75ab	88.49b	24.23b	12.55cb	6.20ab	19.59ab	3.75ab
		2nd year of pot experiment (2024)							
C	0	134.19a	51.15c	170.52a	27.28a	16.03c	4.34c	68.27a	20.16a
S		132.00a	74.35ab	176.91a	28.96a	19.38bc	6.80c	60.20ab	16.95ab
CS150	150	118.49a	56.06bc	123.47bc	24.78a	22.33bc	7.63bc	51.29abc	14.75ab
SS150		126.18a	60.20abc	139.71b	27.44a	25.30abc	9.71bc	55.23abc	15.49ab
CS300	300	119.49a	60.75abc	112.82c	25.98a	36.24ab	14.33ab	40.81c	13.75b
SS300		124.34a	82.01a	138.58b	27.44a	39.97a	17.71a	50.21bc	13.94b
		3rd year of pot experiment (2025)							
C	0	123.96a	44.88ab	185.33a	32.90a	22.13b	7.25b	47.37a	12.89ab
S		130.38a	53.5a	203.22a	36.20a	35.44a	14.4a	51.26a	13.92a
CS150	150	123.25a	36.63b	116.80b	18.10a	13.56b	3.77bc	29.79b	8.46c
SS150		137.25a	49.5a	118.44b	17.70a	14.28b	5.36bc	32.30b	9.01bc
CS300	300	121.38a	33.25b	87.04b	18.73a	9.7b	2.04c	23.30b	5.15c
SS300		124.25a	51.38a	119.91b	18.98a	13.36b	5.16bc	25.84b	6.82c

C - control (0 mM NaCl); S - *Serratia* sp. KF23 (0 mM NaCl); CS150 - salinity (150 mM NaCl); SS150 - salinity + *Serratia* sp. KF23 (150 mM NaCl); CS300 - salinity (300 mM NaCl); SS300 - salinity + *Serratia* sp. KF23 (300 mM NaCl).
In each column, means followed by the same letter are not significantly different at $p \leq 0.05$.

Table 2 Effect of *Serratia* sp. KF23 on the content of chl *a*, chl *b*, total chl, and carotenoids in soybean leaves grown under salinity stress conditions in a three-year (2023, 2024, and 2025) pot experiment

Treatment	NaCl conc. (mM)	Chl *a* (mg/g FW)	Chl *b* (mg/g FW)	Total Chl (mg/g FW)	Carotenoids (mg/g FW)
1st year of pot experiment (2023)					
C	0	0.56a	0.25bc	0.81bc	0.71bc
S		0.58a	0.61a	1.19a	1.25a
CS150	150	0.3c	0.12c	0.41d	0.46c
SS150		0.38b	0.15c	0.54cd	0.55c
CS300	300	0.43b	0.16c	0.58cd	0.64bc
SS300		0.54a	0.39ab	0.93ab	0.87b
2nd year of pot experiment (2024)					
C	0	0.58ab	0.65bc	1.23bc	1.49c
S		0.56b	0.85a	1.41a	1.80b
CS150	150	0.587a	0.64bc	1.22bc	1.89ab
SS150		0.57ab	0.80ab	1.37ab	1.90ab
CS300	300	0.565b	0.56c	1.13c	1.71b
SS300		0.563b	0.96a	1.52a	2.05a
3rd year of pot experiment (2025)					
C	0	0.6a	0.46c	1.06c	1.08c
S		0.579ab	0.72b	1.29b	1.42bc
CS150	150	0.568b	0.82ab	1.39ab	1.64ab
SS150		0.577b	0.80ab	1.38ab	1.56ab
CS300	300	0.576b	0.82ab	1.40ab	1.57ab
SS300		0.557b	1.01a	1.57a	1.91a

C - control (0 mM NaCl); S - *Serratia* sp. KF23 (0 mM NaCl); CS150 - salinity (150 mM NaCl); SS150 - salinity + *Serratia* sp. KF23 (150 mM NaCl); CS300 - salinity (300 mM NaCl); SS300 - salinity + *Serratia* sp. KF23 (300 mM NaCl).
In each column, means followed by the same letter are not significantly different at $p \leq 0.05$.

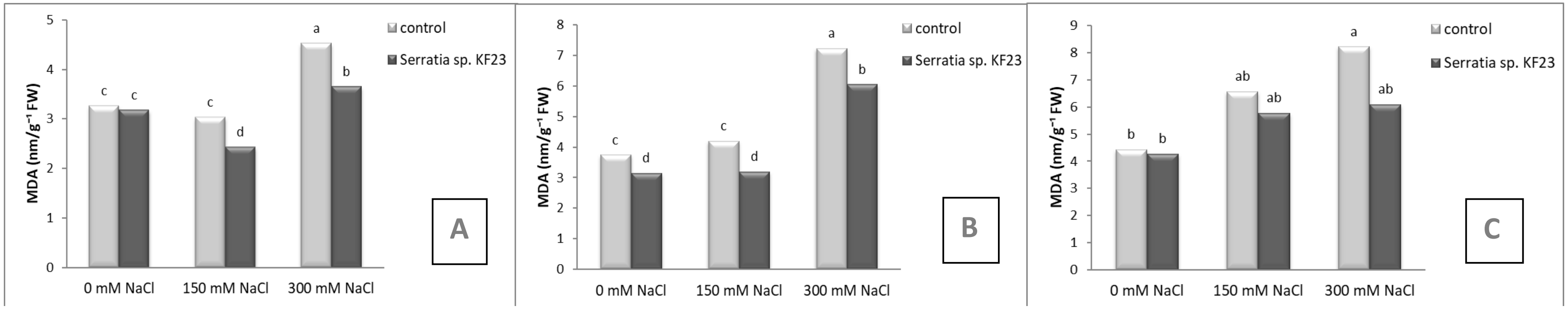

Fig. 1 Effect of *Serratia* sp. KF23 on the content of MDA in soybean leaves grown under salinity stress conditions in a three-year pot experiment a) 2023, b) 2024, and c) 2025. Different letters above the bars indicate significant differences at $p \leq 0.05$.

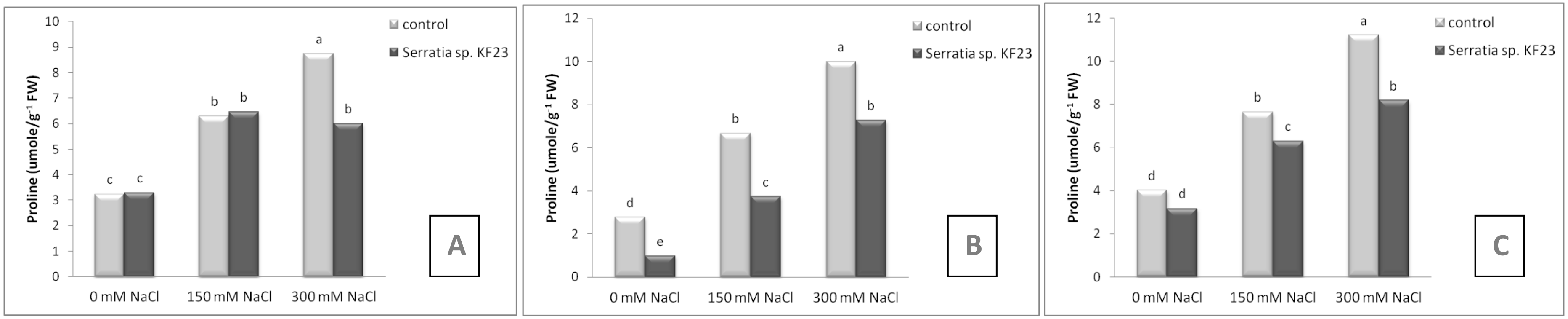

Fig. 2 Effect of *Serratia* sp. KF23 on the content of proline in soybean leaves grown under salinity stress conditions in a three-year pot experiment a) 2023, b) 2024, and c) 2025. Different letters above the bars indicate significant differences at $p \leq 0.05$.

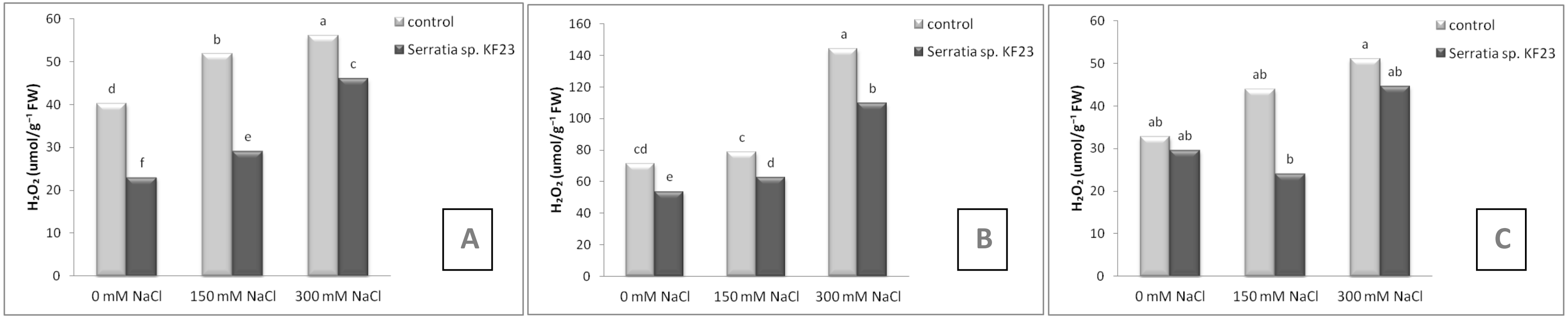

Fig. 3 Effect of *Serratia* sp. KF23 on the content of H_2O_2 in soybean leaves grown under salinity stress conditions in a three-year pot experiment a) 2023, b) 2024, and c) 2025. Different letters above the bars indicate significant differences at $p \leq 0.05$.

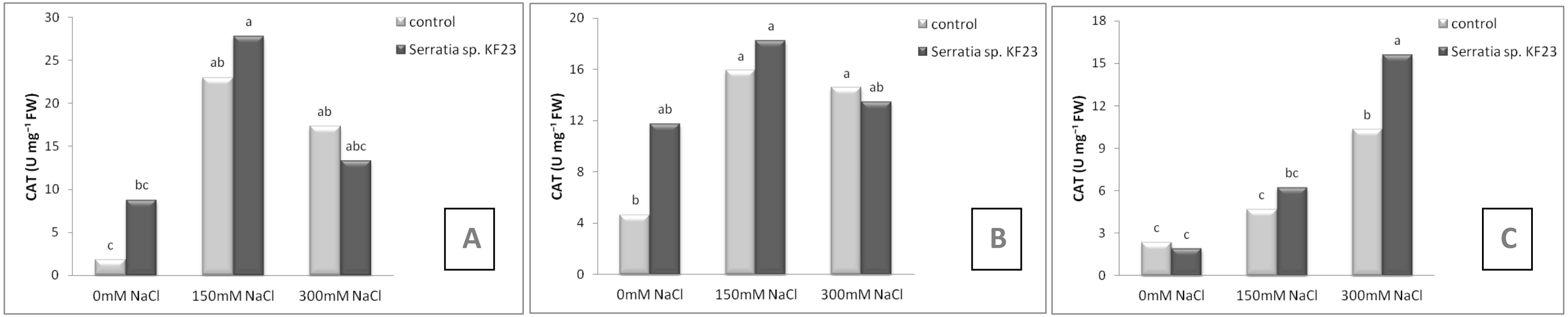

Fig. 4a Effect of *Serratia sp.* KF23 on CAT activity in soybean leaves grown under salinity stress conditions in a three-year pot experiment a) 2023, b) 2024, and c) 2025. Different letters above the bars indicate significant differences at $p \leq 0.05$.

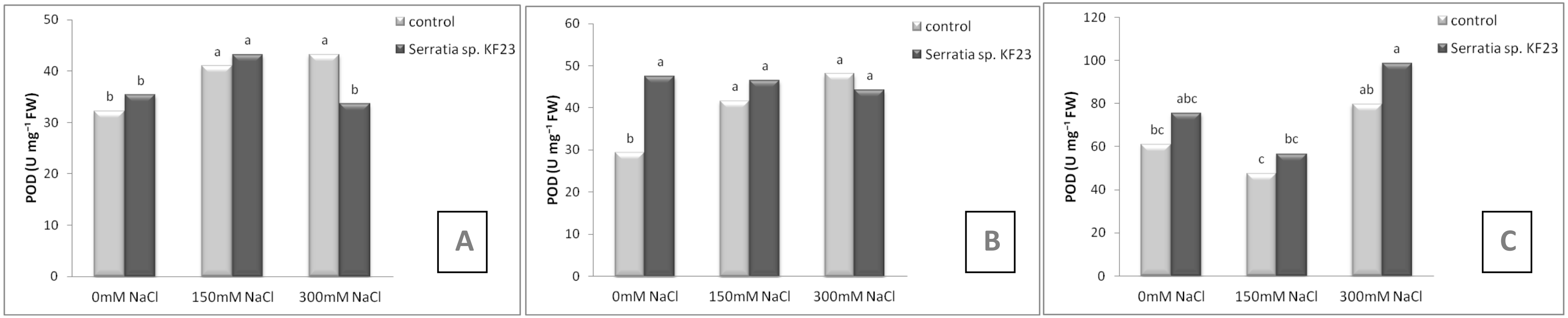

Fig. 5 Effect of *Serratia sp.* KF23 on POD activity in soybean leaves grown under salinity stress conditions in a three-year pot experiment a) 2023, b) 2024, and c) 2025. Different letters above the bars indicate significant differences at $p \leq 0.05$.

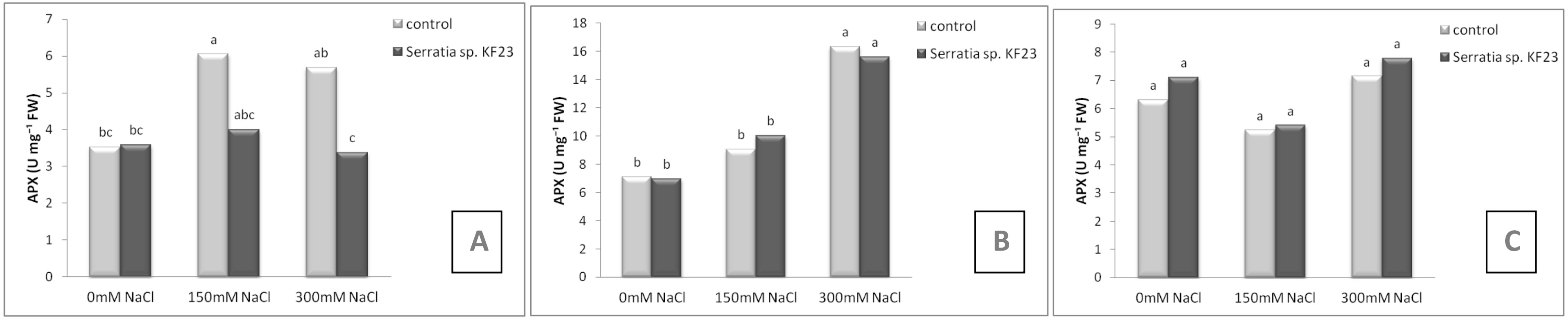

Fig. 6 Effect of *Serratia sp.* KF23 on APX activity in soybean leaves grown under salinity stress conditions in a three-year pot experiment a) 2023, b) 2024, and c) 2025. Different letters above the bars indicate significant differences at $p \leq 0.05$.

Table 3 Physicochemical properties of soil in the pot experiment (2023, 2024, and 2025) as affected by NaCl salinity (0, 150, and 300mM) and *Serratia* sp. KF23 application

Treatments	NaCl conc. (mM)	pH (H_2O)	EC ($dS\ m^{-1}$)	TN (mg/kg^{-1})	K available (mg/kg^{-1} DW)	P available (mg/kg^{-1} DW)	Ca available (mg/kg^{-1} DW)	OC (%)	Humus (% m/m)
		1st year of pot experiment (2023)							
C	0	7.30a	0.72c	2772ab	252.97b	208.04b	1351a	2.31a	3.95a
S		7.28a	0.55c	2816ab	297.17ab	226.93b	1393a	2.39a	3.75a
CS150	150	7.24a	3.68b	2608b	295.65ab	268.12a	1431a	2.28a	4.15a
SS150		7.24a	3.78b	2646b	306.77ab	257.47a	1474a	2.32a	4.13a
CS300	300	7.25a	5.89a	2701ab	328.21a	230.94b	1455a	2.45a	3.85a
SS300		7.26a	5.57a	3009a	283.06ab	230.45b	1576a	2.36a	3.88a
		2nd year of pot experiment (2024)							
C	0	7.45a	0.94c	2772a	766.03a	249a	3286a	2.60a	4.25a
S		7.42a	0.89c	2873a	760.20a	268a	3195a	2.45a	3.9a
CS150	150	7.13b	3.77b	2550ab	646.44a	249a	2802a	2.35a	4.15a
SS150		7.26b	3.74b	2713a	552.11a	236a	2873a	2.45a	4.13a
CS300	300	7.19b	6.66a	2374b	633.06a	246a	2884a	2.5a	3.85a
SS300		7.16b	6.51a	2679ab	716.28a	260a	3082a	2.2a	3.88a
		3rd year of pot experiment (2025)							
C	0	7.00a	0.90c	2773b	623.10bc	314.97a	2841.11ab	2.60a	4.89a
S		7.01a	0.97c	2760b	572.68c	285.05ab	2712.29abc	2.38a	4.33a
CS150	150	6.93a	3.81b	2666b	554.27c	244.40b	2486.36c	2.14a	4.14a
SS150		7.01a	3.62b	2579b	610.73bc	243.41b	2566.58bc	1.91a	3.70a
CS300	300	7.03a	5.72a	3028ab	694.09ab	291.21ab	2767.97abc	2.53a	4.75a
SS300		7.06a	5.75a	3339a	744.99a	294.64ab	2981.99a	2.43a	4.47a

C- control; S- *Serratia* sp. KF23; CS150 - Salinity (150mM NaCl); SS150 - Salinity (150mM NaCl) + *Serratia* sp. KF23; CS300 - Salinity (300mM NaCl); SS300 - Salinity (300mM NaCl) + *Serratia* sp. KF23.

EC - electrical conductivity. TN - total nitrogen. OC - organic carbon.

In each column. means followed by the same letter are not significantly different at $p \leq 0.05$.

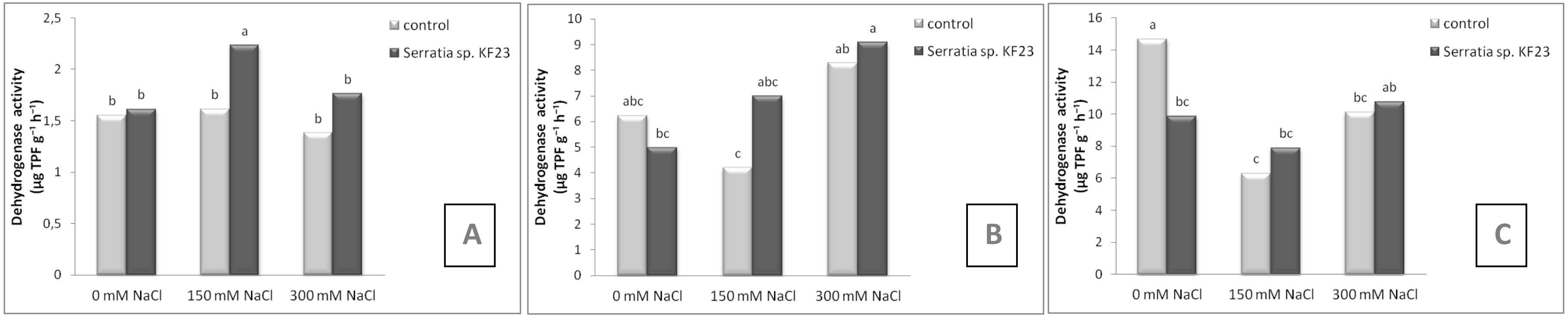

Fig 7a Effect of *Serratia* sp. KF23 on soil dehydrogenase activity under salinity stress in a three-year pot experiment a) 2023, b) 2024, and c) 2025. Different letters above the bars indicate significant differences at $p \leq 0.05$

Table 4 Effect of *Serratia* sp. KF23 on the chemical composition and digestibility of soybean above-ground biomass (excluding pods) under different salinity levels (0, 150, and 300 mM NaCl) in a three-year pot experiment (2023, 2024, and 2025)

Treatment	NaCl conc. (mM)	Total protein (% DM)	Crude fibre (% DM)	Crude ash (% DM)	NDF (% DM)	OM Digestibility (%)	DM Digestibility (%)	WSC (% DM)
	1st year of pot experiment (2023)							
C	0	4.68d	25.14a	7.56b	40.40a	52.42a	49.12a	21.95a
S		7.18c	26.03a	7.42b	40.93a	53.73a	50.66a	20.1a
CS150	150	7.76bc	28.96a	13.79a	41.77a	43.88a	44.37a	6.93b
SS150		8.15bc	30.61a	13.25a	43.68a	43.93a	44.55a	7.14b
CS300	300	9.85ab	27.67a	14.35a	40.54a	47.09a	46.75a	6.39b
SS300		11.06a	26.81a	14.82a	38.53a	52.35a	52.91a	7.57b
2nd year of pot experiment (2024)								
C	0	6.47b	33.39a	9.31c	47.45a	38.79a	38.37a	9.37a
S		7.31b	31.68a	9.49c	45.49a	41.09a	40.58a	10.00a
CS150	150	10.59ab	27.22a	17.84a	42.89a	36.76a	35.29a	2.41b
SS150		10.50ab	29.05a	15.94ab	44.65a	36.98a	35.82a	3.97ab
CS300	300	10.80ab	28.64a	14.3b	43.46a	41.59a	39.82a	4.74ab

SS300		12.97a	26.37a	14.80b	41.69a	48.09a	46.03a	5.52ab
3rd year of pot experiment (2025)								
C	0	6.47c	29.95a	7.89c	44.03a	45.31a	43.99a	15.09a
S		9.43bc	28.33a	8.65c	42.68a	49.33a	48.93a	14.48a
CS150	150	9.84b	28.33a	11.22b	42.53a	47.33a	47.12a	11.26ab
SS150		10.09b	30.04a	11.30ab	43.92a	43.48a	44.09a	8.48b
CS300	300	10.96b	31.00a	12.19ab	44.78a	41.87a	42.45a	7.20b
SS300		14.70a	25.88a	12.35a	39.64a	50.79a	50.43a	8.34b

C - control (0 mM NaCl); S - *Serratia* sp. KF23 (0 mM NaCl); CS150 - salinity (150 mM NaCl); SS150 - salinity + *Serratia* sp. KF23 (150 mM NaCl); CS300 - salinity (300 mM NaCl); SS300 - salinity + *Serratia* sp. KF23 (300 mM NaCl).

NDF - Neutral Detergent Fiber. OM Digestibility- Organic Matter Digestibility. DM Digestibility - Dry Matter Digestibility. WSC - total soluble sugars.

In each column. means followed by the same letter are not significantly different at $p \leq 0.05$.
